# Integrating multiple chemical tracers to elucidate the diet and habitat of Cookiecutter Sharks

**DOI:** 10.1038/s41598-021-89903-z

**Published:** 2021-06-03

**Authors:** Aaron B. Carlisle, Elizabeth Andruszkiewicz Allan, Sora L. Kim, Lauren Meyer, Jesse Port, Stephen Scherrer, John O’Sullivan

**Affiliations:** 1 School of Marine Science and Policy, University of Delaware, Lewes, DE USA; 2 Department of Civil and Environmental Engineering, Stanford University, Palo Alto, CA USA; 3 Department of Life and Environmental Sciences, University of California Merced, Merced, CA USA; 4 Southern Shark Ecology Group, College of Science and Engineering, Flinders University, Adelaide, SA Australia; 5 Georgia Aquarium, Atlanta, GA USA; 6 Center for Ocean Solutions, Stanford University, Monterey, CA USA; 7 Department of Oceanography, University of Hawaii, Honolulu, HI USA; 8 Monterey Bay Aquarium, Monterey, CA USA; 9 Present Address: School of Marine and Environmental Affairs, University of Washington, Seattle, USA

**Keywords:** Marine biology, Stable isotope analysis, Ecology, Food webs, Environmental chemistry

## Abstract

The Cookiecutter shark (*Isistius brasiliensis*) is an ectoparasitic, mesopelagic shark that is known for removing plugs of tissue from larger prey, including teleosts, chondrichthyans, cephalopods, and marine mammals. Although this species is widely distributed throughout the world’s tropical and subtropical oceanic waters, like many deep-water species, it remains very poorly understood due to its mesopelagic distribution. We used a suite of biochemical tracers, including stable isotope analysis (SIA), fatty acid analysis (FAA), and environmental DNA (eDNA), to investigate the trophic ecology of this species in the Central Pacific around Hawaii. We found that large epipelagic prey constituted a relatively minor part of the overall diet. Surprisingly, small micronektonic and forage species (meso- and epipelagic) are the most important prey group for Cookiecutter sharks across the studied size range (17–43 cm total length), with larger mesopelagic species or species that exhibit diel vertical migration also being important prey. These results were consistent across all the tracer techniques employed. Our results indicate that Cookiecutter sharks play a unique role in pelagic food webs, feeding on prey ranging from the largest apex predators to small, low trophic level species, in particular those that overlap with the depth distribution of the sharks throughout the diel cycle. We also found evidence of a potential shift in diet and/or habitat with size and season. Environmental DNA metabarcoding revealed new prey items for Cookiecutter sharks while also demonstrating that eDNA can be used to identify recent prey in stomachs frozen for extended periods. Integrating across chemical tracers is a powerful tool for investigating the ecology of elusive and difficult to study species, such as meso- and bathypelagic chondrichthyans, and can increase the amount of information gained from small sample sizes. Better resolving the foraging ecology of these mesopelagic predators is critical for effective conservation and management of these taxa and ecosystems, which are intrinsically vulnerable to overfishing and exploitation.

## Introduction

Knowledge of the ecological role of deep water chondrichthyans is needed as these species and ecosystems are increasingly being exploited by fisheries and other extractive industries^1^. This is particularly problematic as the chondrichthyans inhabiting these environments are known to be inherently vulnerable to overexploitation due to their conservative demographic characteristics and low population rebound potential^2^. As meso- and upper trophic level predators, elasmobranchs play an important role in deep water oceanic ecosystems^3^, yet lack of data on their trophic ecology in oceanic ecosystems hinders effective management of these vulnerable species and the ecosystems of which they are part.

The diets and ecological roles of many meso- and bathypelagic elasmobranchs, and indeed most deep-water predators, are poorly described due to the difficulty of accessing animals and because many, often the majority sampled, have empty stomachs^3–5^. Furthermore, when present, identification of prey items is hindered by their state of digestion and high degree of fragmentation^4^. This circumstance has led to the diets of many deep-water species being based on very limited information. For example, Cortés^6^ reported that 66% of reported Squaliform shark diets (n = 32 spp.), which are primarily deep-water species, were based on sample sizes < 100 and 41% were based on < 20 stomachs, numbers that are hardly adequate for describing their diet, especially considering some of the limitations of stomach content analysis (e.g. empty stomachs provide no data, contents reflect a snap shot of diet, need for large sample sizes, etc.).

One approach to improving our understanding of the trophic ecology of these species, and other poorly studied, difficult to access species, is to use approaches that are complementary to traditional stomach content analyses yet avoid some of their issues. Chemical tracer approaches complement traditional diet analyses in that there are no “empty stomachs”, dietary information includes long-term feeding behavior (integrated over different temporal scales depending on tissue type), and dietary information reflects assimilated prey, not just ingested prey^7–9^. While small sample sizes are still limiting, much can still be learned and some of the limitations of small sample size can potentially be overcome by using multiple tracer techniques.

Recent analytical and technological advances have led to an increase in the use of biochemical tracer approaches, including stable isotope analysis (SIA) and fatty acid analysis (FAA), to assess the diet and habitat use of animals based on the chemical composition of their tissues^10–12^. SIA uses the stable isotope composition (i.e., the ratio of heavy isotopes to light isotopes) of tissues to provide information on the ecology an organism^13–15^. These inferences are possible because the stable isotope composition of an organism is directly related to that of its prey. The isotopic composition of consumers shifts in a predictable manner with isotopic fractionation of ^13^C/^12^C and ^15^N/^14^N increasing with trophic level^7^. The stepwise enrichment in ^13^C and ^15^N with increasing trophic level is known as a trophic discrimination factor (TDF)^16,17^. The metabolic activity of different tissues influences the isotopic incorporation rate of tissue (i.e., turnover)^18^, meaning that different tissues integrate dietary information over different amounts of time. More metabolically active tissues, such as plasma, red blood cells, or liver, incorporate dietary information over weeks to months, whereas muscle integrates diet over many months to > 1 year^19–21^. Hence, stable isotope analysis of different tissues provides integrated estimates of diet over different temporal scales and can be used to understand temporal shifts in diet or habitat^22,23^.

The use of fatty acid analysis (FAA) in elasmobranch trophic ecology is growing^11,12^, owing to its ability to trace diet and habitat use at finer resolution than other biochemical methods. Distinct chemical structures are retained from different basal food-chain production (e.g., bacteria, diatoms, dinoflagellates) (^24–26^) and these compounds are passed through the food web with minimal modification. This transfer allows feeding ecology to be traced across different habitats with distinct basal sources, including differentiating epipelagic and meso- and bathypelagic prey^11^. Furthermore, certain FAs are preferentially assimilated into distinct taxa-specific tissues (i.e., teleost muscle vs. marine mammal blubber vs. cephalopod muscle), providing insight into key prey items when investigating predator diets^27–29^. Fatty acids are more metabolically active than bulk protein, so they reflect changes in diet at shorter time scales than stable isotopes (3–16 weeks vs. 6 months–1 year for elasmobranch muscle)^30^. These characteristics make FAA an ideal tool to explore recent feeding ecology^31^, including ontogenetic^32^ or spatial–temporal shifts in diet^33^ with a high degree of detail.

Researchers have also used genetic approaches for diet analysis by identifying environmental DNA (eDNA) from stomach contents of organisms^34^. While eDNA metabarcoding has been more commonly used on feces or scat samples^35–37^, recent studies have used this method to investigate the stomach contents of marine organisms, including chondrichthyan fishes^38^. To identify prey species using eDNA metabarcoding, DNA is extracted from stomach contents and then amplified by polymerase chain reaction (PCR) using universal or group-specific primer sets designed to identify DNA from a mixture with multiple species. Once the eDNA is amplified, it is sequenced using high-throughput sequencing technologies (e.g., Illumina MiSeq or HiSeq), and representative sequences are compared to a database of known organisms for identification. In addition to metabarcoding, quantitative PCR (qPCR) can be used for species-specific eDNA investigations of stomach contents^39^ if a particular prey is known a priori. Unlike metabarcoding, which is best suited to answer questions related to the presence/absence or relative abundance of species, qPCR is more quantitative and can provide an estimate of the total sample target DNA concentration, but only provides information of the single species of interest.

The Cookiecutter shark (*Isistius brasiliensis*, Quoy and Gaimard, 1824, Squaliformes: Dalatiidae) is a small (up to ~ 50 cm total length, TL), common mesopelagic predator that, while widely distributed throughout the world’s tropical and subtropical oceanic waters, has been little studied, as is the case for many deep water, oceanic predators. The shark has a broad depth distribution, ranging from the surface to depths in excess of 1500 m^40,41^. Although Cookiecutter sharks are known to inhabit mesopelagic habitats, they have generally only been observed near surface waters during the night, leading researchers to believe that they exhibit diel vertical migration^42,43^.

Despite its wide distribution and apparent abundance in oceanic habitats around the world, Cookiecutter sharks are very difficult to access and study, and as a result, much about the biology and ecology of this species is poorly known^44,45^. A unique aspect of Cookiecutter shark biology is its mode of feeding. The jaws, dentition, and tongue morphology allow Cookiecutter sharks to remove plugs of tissue from larger animals, including teleosts, elasmobranchs, marine mammals, marine reptiles, and cephalopods^41,46,47^. Widder^48^ hypothesized that the sharks use an ambush style of hunting where individuals sit and wait for passing prey. Once Cookiecutter sharks are close enough, they latch onto their prey, spin, and remove large plugs of tissue. This ectoparasitic mode of foraging leaves distinctive scars and markings on prey. Many details about the distribution and ecology of both Cookiecutter sharks and their prey are based on the ease of observing these markings, which are frequently observed due to their occurrence on large charismatic and economically important species^45,49,50^.

Large prey are generally assumed to be an important part of Cookiecutter shark diet due to the frequency of bite observations on large species; however, actual dietary information for this species remains surprisingly limited, with only four published studies to date that examine stomach contents of Cookiecutter sharks^41,47,51,52^. The samples for these studies were collected at different times between 1951 and 2015 across the Indian, Pacific, and Atlantic Oceans. In total, 147 stomachs were examined, of which 83 (56%) had contents within them and 64 (44%) were empty. In terms of general prey types, stomachs included ~ 49% cephalopods (beaks, tissues), ~ 23% fish (tissue plugs, whole), ~ 25% marine mammal remains (tissue plugs, skin, blubber), and ~ 3% crustacean parts. Although it appears that sharks will consume small squid whole, many of the squid remains were estimated to be from relatively large squid, often as large as the sharks themselves. Given the lack of empirical data on the diet of Cookiecutter sharks, we only have the most basic understanding of their trophic ecology with little to no information on size, temporal, or spatial variability in diet. In addition, due to the high proportion of empty stomachs encountered in these studies, and the historical difficulty of identifying the origin of the various tissue plugs in shark stomachs, traditional approaches to assessing diet (e.g., stomach content analysis) have been of reduced utility.

The goal of this study was to use multiple biochemical tracer approaches (SIA, FAA, and eDNA) to elucidate the trophic ecology of Cookiecutter sharks in the Central Pacific and demonstrate the utility of this integrated approach for investigating the trophic ecology of difficult to study species, where sample sizes are often limited. In particular, we were interested in using SIA and FAA to assess the relative importance of different prey groups (mesopelagic, epipelagic, and vertically migrating prey) to Cookiecutter sharks across a size range. We also investigated the utility of using eDNA approaches to identify prey from Cookiecutter shark stomachs.

## Results

### Stable isotope analysis: comparisons of size and sex

**Figure 1:**
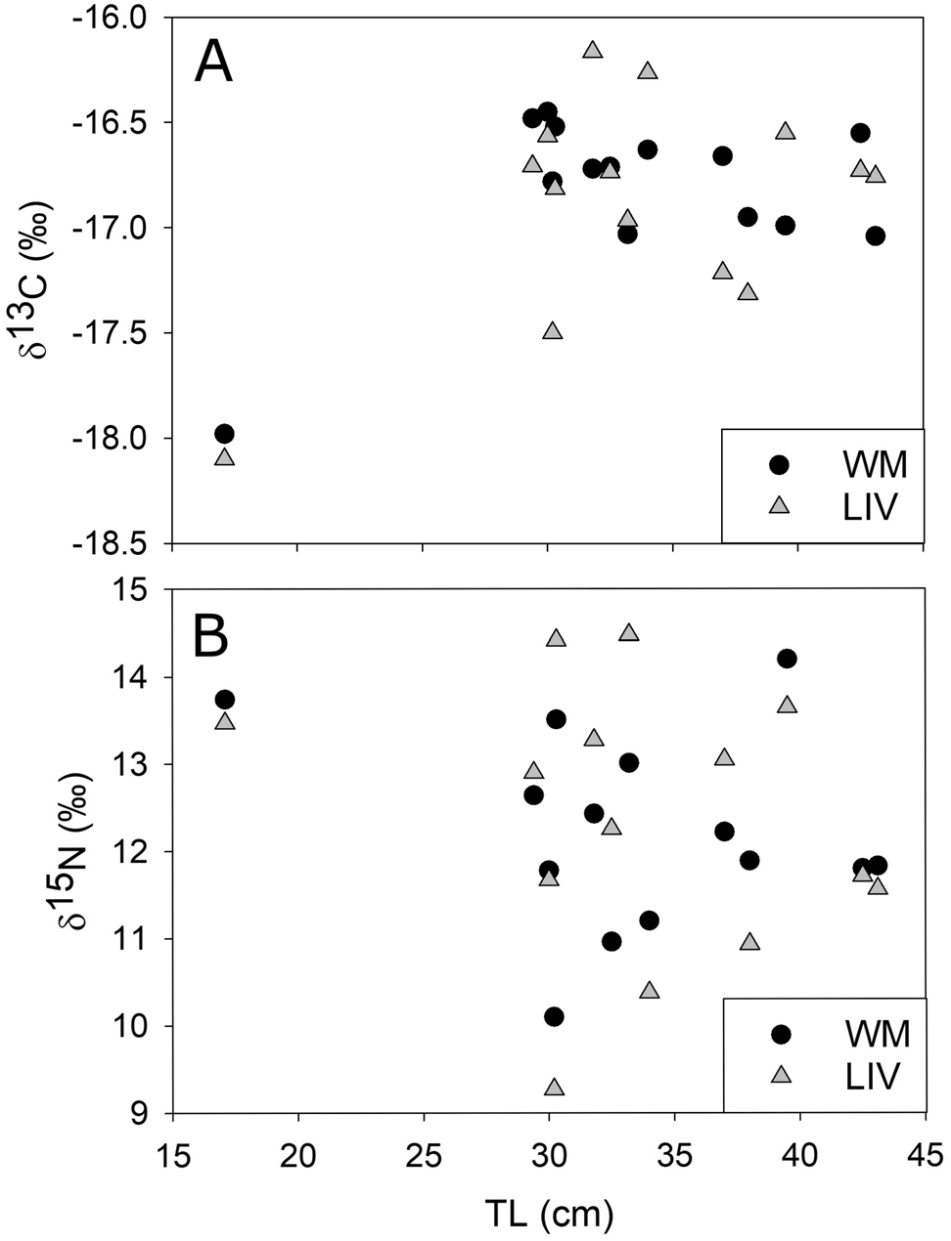
Carbon (**A**) and nitrogen (b) stable isotope composition of Cookiecutter shark tissues (*WM* white muscle, *LIV* liver) by length. There was no significant relationship (linear regression, p > 0.05) between δ^13^C and δ^15^N and length for white muscle (solid lines, δ^13^C r^2^ = 0.12, δ^15^N = 0.04) and liver (dashed lines, δ^13^C r^2^ = 0.15, δ^15^N = 0.04).

There was no significant relationship between length and δ^13^C or δ^15^N values (Fig. 1) for liver (linear regression, δ^13^C: r^2^ = 0.22, p > 0.09; δ^15^N: r^2^ = 0.03, p > 0.54) or muscle (linear regression, δ^13^C: r^2^ = 0.17, p > 0.15; δ^15^N: r^2^ = 0.04, p > 0.51). However, the smallest shark (17.1 cm TL) had one of the highest δ^15^N and lowest δ^13^C values of all sharks for both liver and muscle. There was no significant difference between sexes in muscle (δ^13^C: Mann–Whitney Rank Sum Test, p = 0.38; δ^15^N: T-test, p = 0.39). In liver, there was no significant difference in δ^15^N (t test, p = 0.12) though there was in δ^13^C (T test, p = 0.03), with females having higher δ^13^C than males. However, low samples sizes limit the power of these tests.

### Stable isotope analysis: prey contributions

Potential Cookiecutter shark prey groups generally clustered based on their habitat type (see “Methods”). One group (hereafter “DVM group”), was primarily comprised of large species that exhibit diel vertical migration as well as marine mammals. Another group (“MESO group”), was entirely comprised of small mesopelagic and epipelagic micronektonic and forage species. The last group (“EPI group”), was primarily made of large epipelagic species.

**Figure 2:**
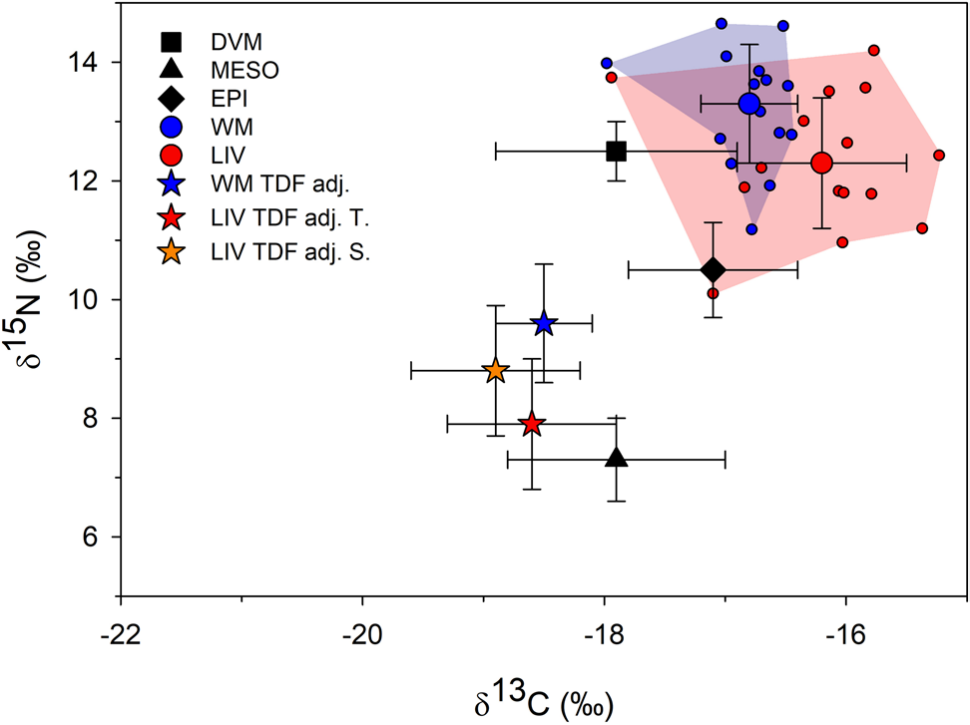
Mean (SD) δ^13^C and δ^15^N of white muscle (WM, blue circles) and liver (LIV, red circles) of Cookiecutter sharks and primary prey groups (black symbols). Blue and red circles show data for individual sharks, with the minimum convex polygons (MCP) for each tissue being shown by the polygons. The mean values for shark tissues were adjusted down one trophic level to account for trophic discrimination factors (TDFs) based on Kim et al.^53^ for WM (blue star) and TDFs from this study for LIV based on Tilapia (red star, LIV TDF adj. T.) and squid (orange star, LIV TDF adj. S.).

**Figure 3:**
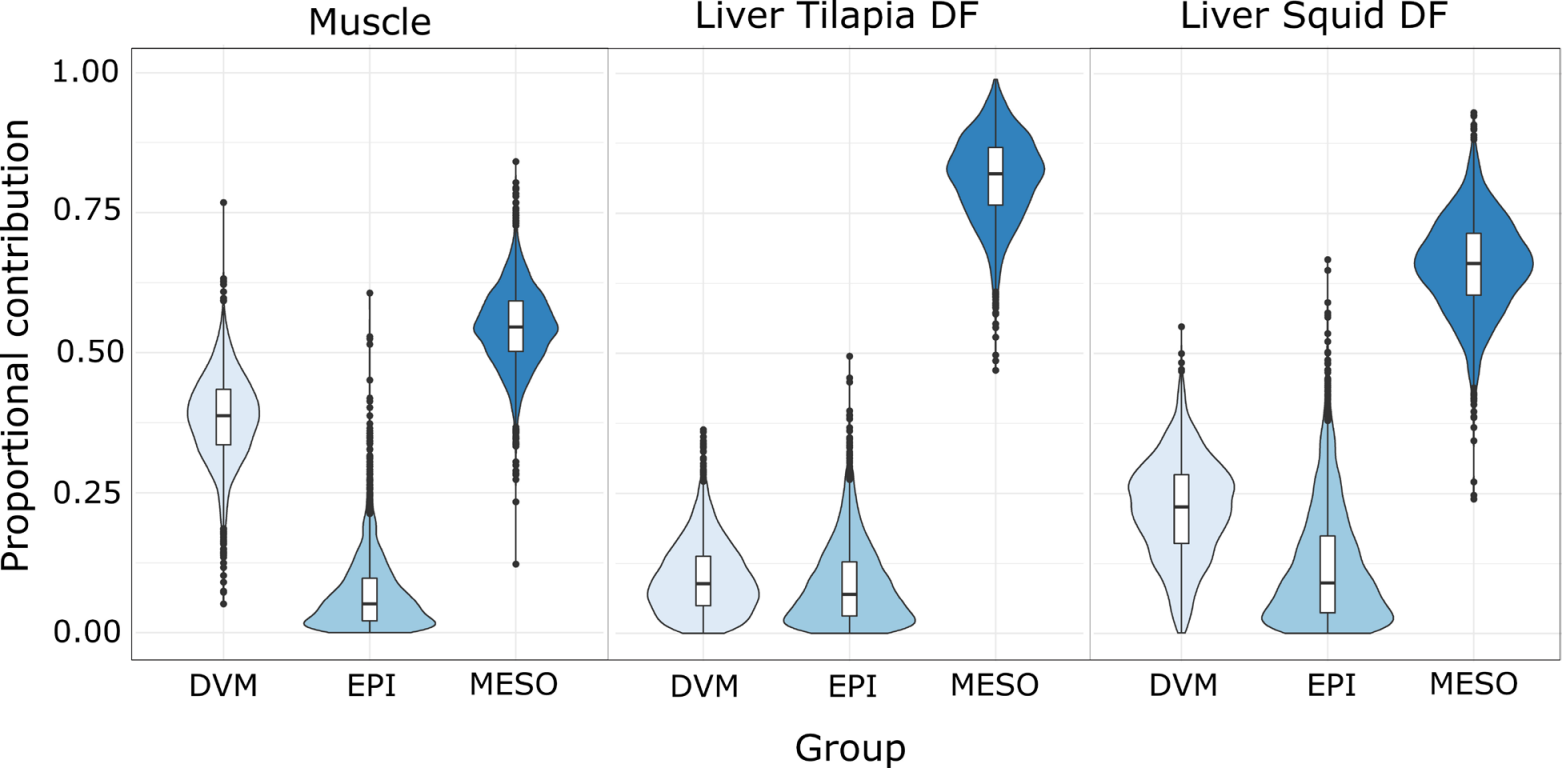
Estimated proportional contribution of different prey groups to Cookiecutter shark white muscle and liver using MixSIAR.

A direct comparison of Cookiecutter shark (Table 1) and potential prey stable isotope values (not accounting for TDF) show that δ^13^C values of shark muscle were similar to the EPI values and generally higher than those of the prey other groups, whereas δ^15^N values of both tissues were higher than those of EPI, but similar to DVM values (Fig. 2, Table 1). When muscle was adjusted down one trophic level to account for TDF, adjusting the consumer to resemble potential prey, using TDFs of 1.7 ± 0.5 for δ^13^C and 3.7 ± 0.5 for δ^15^N^53^, δ^13^C values align with DVM and MESO groups, whereas δ^15^N values are intermediate to these two groups. Mixing model results for muscle suggest that Cookiecutter sharks primarily feed upon DVM and MESO prey (or prey with isotopic composition similar to these groups) (Fig. 3). The estimated median proportional contribution of MESO prey was 55% (40–68% 95% credible intervals), while DVM prey was 39% (22–53%) and EPI was 5% (0–2%).

**Table 1:** Mean (SD) δ^13^C and δ^15^N for Cookiecutter shark tissues (bulk: lipid and urea extracted samples), and tissues adjusted down one trophic level to account for trophic discrimination factors (TDF).

	δ13C		δ15N		C:N	
	Mean	SD	Mean	SD	Mean	SD
**Muscle**						
Bulk	− 16.8	0.4	13.3	1.0	3.1	0.0
TDF adj	− 18.5	0.4	9.6	1.0		–
**Liver**						
Bulk	− 16.2	0.7	12.3	1.1	3.5	0.4
TDF adj. T	− 18.6	0.7	7.9	1.1	–	–
TDF adj. S	− 18.9	0.7	8.8	1.1	–	–

Liver samples were adjusted based on TDFs from Tilapia (T) and squid (S) diets (see “Methods”).

Because there is a lack of TDFs for elasmobranch liver from controlled feeding studies, we used liver samples that had been archived from a previous study that characterized TDFs for different Leopard Shark (*Triakis semifasciata*) tissues^53^. We estimated TDFs for Leopard Shark liver fed on diets of squid (*Doryteuthis opalescens*) or Tilapia (*Oreochromis aureus*) (see Supplemental Information for full details on TDF estimates). Leopard Sharks fed squid had a Δ^13^C_squid_ (TDF) of 2.7‰ ± 0.5 and a Δ^15^N_squid_ of 3.5‰ ± 0.7 with error propagation. Sharks fed tilapia had a Δ^13^C_Tilapia_ = 2.4‰ ± 0.9 and Δ^15^N_Tilapia_ = 4.4‰ ± 0.4 with error propagation. Liver mixing model results indicated increased use of MESO prey relative to muscle using TDFs from both squid and Tilapia. The estimated median proportional contribution of MESO prey was 66% (47–82% credible intervals), while DVM prey was 23% (4–39%) and EPI was 9% (0–38%) using squid TDFs. The estimated median proportional contribution of MESO prey was 82% (65–95%), while DVM prey was 9% (1–24%) and EPI was 7% (0–27%) using Tilapia TDFs. The major difference between muscle and liver mixing model results was that liver, which has a shorter incorporation rate than muscle, showed higher contribution of MESO prey and reduced DVM relative to muscle for both squid and Tilapia TDFs.

### Fatty acid analysis: comparisons of size and sex

Muscle and liver FA profiles (Appendix S1: Tables S2, S3) did not differ by sex (PERMANOVA P(MC) = 0.495 and 0.353 respectively), yet size was a significant driver of muscle FA profiles [main test P(MC) = 0.003]. The large sharks were distinguished by higher levels of 18:1ω9, and 18:1ω7 (Figs. S2, S3), most similar to Squaliform, Hexanchiform, and Mako Sharks, while the smallest shark (Figures S2, S3) contained high levels of 20:5ω3, 22:5ω3 and 20:0 compared to the other Cookiecutter sharks (Fig. S2).

### Fatty acid analysis: foraging habitats

**Figure 4:**
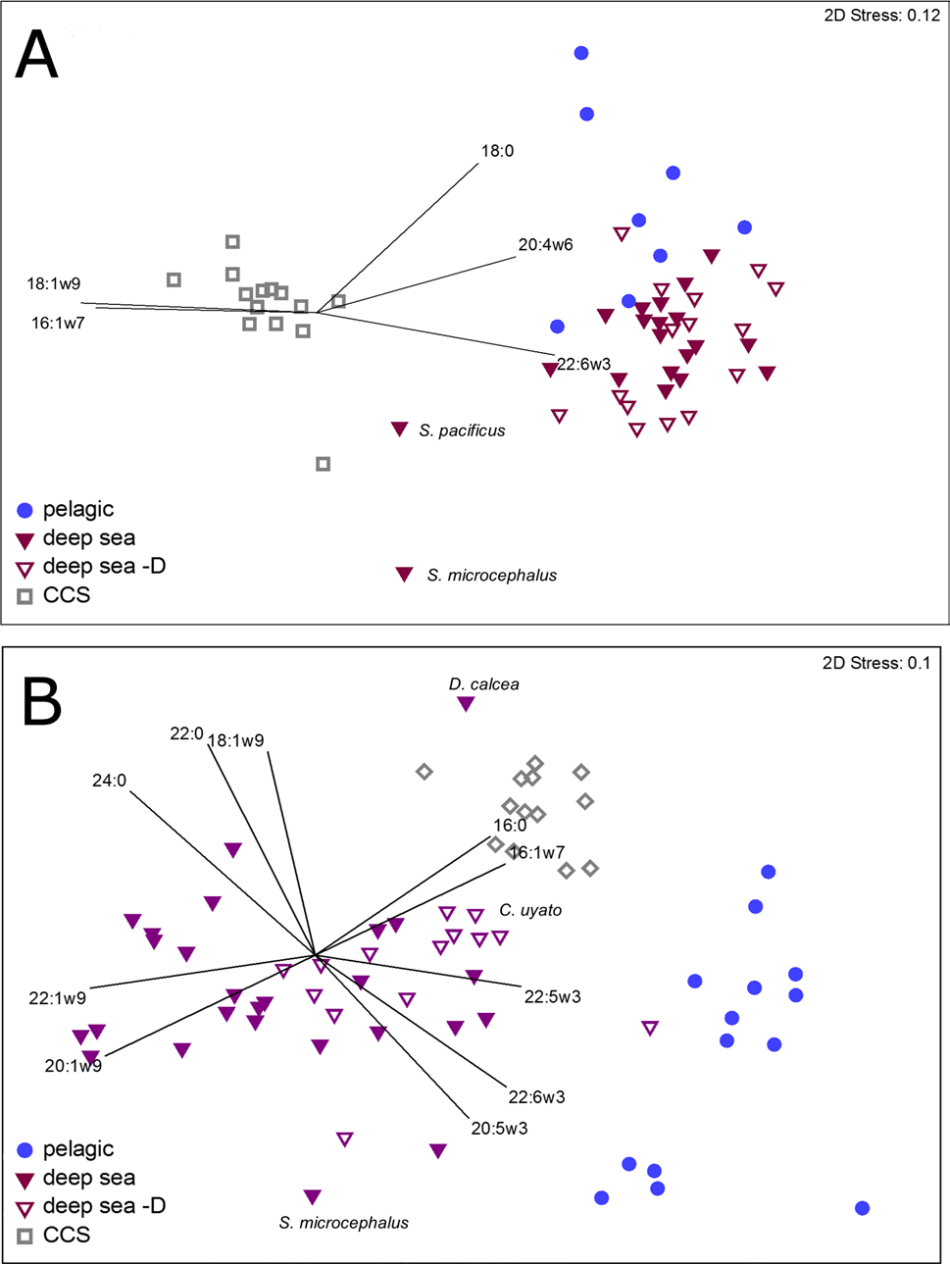
Non-metric MDS of (**A**) chondrichthyan muscle FA profiles (% contribution) and (**B**) liver FA profiles with vector overlays showing FAs with correlation values > 0.8. The results are displayed by habitat type (pelagic, deep sea, and deep sea demersal [indicated by –D], Appendix S1: Table S4), with the exception of the Cookiecutter sharks (CCS). *S. pacificus: Somniosus pacificus, S. microcephalus: Somniosus microcephalus, D. calcea: Deania calcea, C. uyato: Centrophorus uyato*.

Habitat type was a significant driver of muscle FA profiles [main test P(MC) = 0.001], with respect to each of the three habitat types (pelagic, deep sea, and deep sea demersal, Tables S2–S4). The Cookiecutter sharks differed substantially from all other chondrichthyans group by habitat (all pairwise test P(MC) values < 0.05, Fig. 4) suggesting unique prey/habitat preferences. The Cookiecutter sharks were most similar to deep-sea sharks, followed by the pelagic and deep-sea demersal chondrichthyans (SIMPER dissimilarity scores 31.76, 32.75 and 34.57 respectively, Fig. 4, Fig. S4). Cookiecutter shark muscle had extremely high levels of 18:1ω9 (44.48 ± 3.64, Table S2, Fig. S3) and 16:1ω7 (7.50 ± 1.30, three times greater than the other three habitat groups; Table 2, Figs. S3, S5). These high levels of 18:1ω9 and 16:1ω7 drove the similarity between Cookiecutter sharks, Pacific Sleeper Sharks *Somniosus pacificus*, and Greenland Sharks *Somniosus microcephalus* (Fig. 4), and Mako Sharks, *Isurus oxyrinchus* (Table S3, Fig. S3). Cookiecutter sharks had low levels of essential FA 22:6ω3, with only 3.4 ± 0.7% contribution versus 19.2 ± 6.5% in pelagic, 28.8 ± 6.8% in deep sea demersal, and 29.0% ± 9.4% in deep sea chondrichthyans (Table 2, Fig. S5).

**Table 2:** SIMPER output of relative dissimilarity scores between Cookiecutter sharks (CCS) muscle FA profiles and prey group FA profiles.

Prey groups	Dissimilarity	FAs (habitat containing the highest amount)
CCS—DVM/MESO	27.40	22:6ω3 (DVM), 18:1ω9 (CCS), 20:5ω3 (DVM), 16:1ω7 (CCS)
CCS—small MESO/EPI	28.31	22:6ω3 (SM), 18:1ω9 (CCS), 20:5ω3 (SM), 16:1ω7 (CCS)
CCS—mammal	28.52	18:1ω9 (CCS), 20:1ω9 (MM), 16:0 (CCS), 20:5ω3 (MM)
CCS—EPI big	34.43	18:1ω9(CCS) , 22:6ω3 (EPI), 16:1ω7 (CCS), 22:5ω3(EPI)
CCS—cephalopods	36.64	18:1ω9 (CCS), 22:6ω3 (EPI), 20:5ω3(CP), 20:1ω9 (CCS)

The top two FAs driving the dissimilarity are ordered by contribution, with the group containing the highest amount indicated in parenthesis.

Habitat type was also the significant driver of liver FA profiles (main test P(MC) = 0.001, all pairwise tests P(MC) values < 0.001, Fig. 4, Appendix S1: S3, Table S3, S5) but unlike the muscle (Table S2), liver FA profiles were most similar to deep sea demersal, followed by the pelagic and then the deep sea chondrichthyans (SIMPER dissimilarity scores 17.48, 20.63 and 23.23 respectively). Cookiecutter sharks were most like Little Gulper Sharks, *Centrophorus uyato*, and Birdbeak Dogfish, *Deania calcea*. As with the muscle profiles, Cookiecutter livers were extremely high in 18:1ω9, and relatively high levels of 16:0 and 16:1ω9, which drove the similarities between the Cookiecutter and Gulper Sharks and Birdbeak Dogfish (Fig. 4).

### Fatty acid analysis: comparison to potential prey items

**Figure 5:**
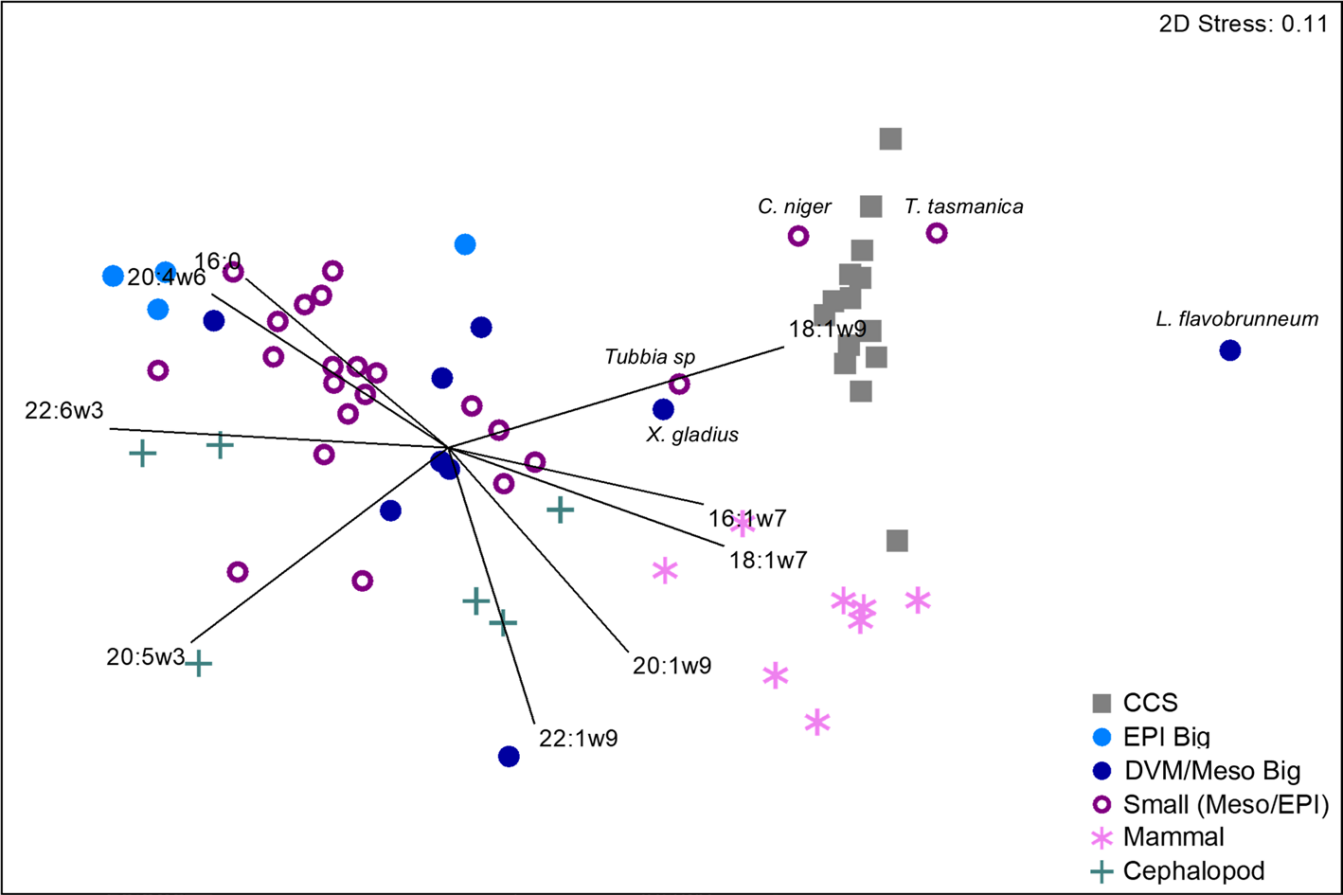
Non-metric MDS of Cookiecutter shark muscle and liver (grouped, CCS) and potential prey item FA profiles (% contribution). Vector overlays show FAs with PCO correlation values > 0.8.

Each prey classification, including the Cookiecutter sharks, had distinct FA profiles (main test P(MC) = 0.001, all pairwise test P(MC) values < 0.05). The Cookiecutter shark profiles were most similar to the DVM teleosts (SIMPER relative dissimilarity of 27.40) followed by the MESO teleosts (28.31) and marine mammals (28.52), and were most different from the EPI teleosts (34.43) and cephalopods (36.64) (Fig. 5, Figs. S1–S3, Table S6). On a species level, MESO rudderfish *Tubbia* spp. and medusa fish *Centrolophus niger,* and DVM Swordfish *Xiphias gladius* and escolar *Lepidocybium flavobrunneum* from around Tasmania, Australia (Table S9) were most similar in FA profiles to the Cookiecutter sharks, due to particularly high 18:1ω9 levels.

### Environmental DNA (eDNA)

Of the 14 total sharks, 10 sharks had target DNA from gastric fluid successfully amplified and sequenced using eDNA metabarcoding. Visible tissue of prey was present in three out of these 10 stomachs, and these tissue samples were Sanger sequenced (Fig. S6). After merging paired-end reads, fastq quality filtering, identifying tags and adapters, and removing singletons, 8,401,894 high quality reads remained in the stomach samples, positive controls, and negative controls. The average number of reads per stomach sample was 227,505 and ranged from 839 to 321,355. The two extraction blanks had 0 and 53,815 reads respectively; these reads were annotated as human, chimeric sequences (i.e., artifact sequences formed during PCR when two or more biological sequences incorrectly join) or were unassigned based on no matches to our 12S database. The reads from the stomach samples and positive controls were rarefied to 100,000 per sample to account for unequal sequencing depths. All subsequent results presented below are based on the rarefied dataset.

The three replicates of the Swordfish control produced a total of 299,990 out of 300,000 reads assigned to Swordfish, indicating negligible cross contamination across the samples. Sequencing of the mock community replicates identified 10 out of the 10 mock community taxa; there were no more than 5 reads assigned to taxa not present in the mock community. A total of 79,068 reads could not be taxonomically assigned to our 12S database or to a taxonomic rank within MEGAN, however these unassigned results represent just 2.8% of the rarefied reads. Of the 2,800,000 total reads in the rarefied dataset, 1,186,484 (42.4%) were assigned to Cookiecutter shark.

We identified three prey in the stomach samples of the 10 sharks that were processed: *Ariomma* sp. (genus of deep-water fish), *Cololabis saira* (Pacific saury) and the tribe *Thunnini* (tunas). These three taxa accounted for 93.2% of the rarefied reads when reads assigned to Cookiecutter shark are excluded. Within the 10 stomachs, *Ariomma* were detected in five, *Cololabis* were detected in two, and *Thunnini* were detected in four individuals (Table 3). Sanger sequencing of the tissue contents present in three stomachs confirmed the presence of *Cololabis* and *Thunnini.* The maximum number of prey detected in any stomach was two. Tunas were annotated to the tribe level of *Thunnini* because MEGAN was unable to identify the resolution at the genus level due to similarities between genera in the short fragment amplified. However, *Thunnus* and *Auxis*, two genera within *Thunnini*, were identified in the dataset and both these genera have geographic distributions within the Hawaiian Islands.

**Table 3:**
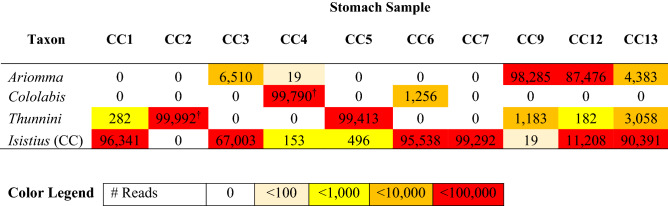
Heatmap of prey identified in the stomachs of Cookiecutter sharks (CC) via eDNA metabarcoding of gastric fluid contents.

Data for a given stomach sample (e.g. CC1 is Cookiecutter shark one) is presented as the mean read count from the rarefied dataset. Note that while there was not sufficient DNA for metabarcoding of CC11, a tissue sample from this stomach was Sanger sequenced and identified as *Thunnini.* ^†^Confirmed by Sanger sequencing of tissue contents.

## Discussion

**Figure 6:**
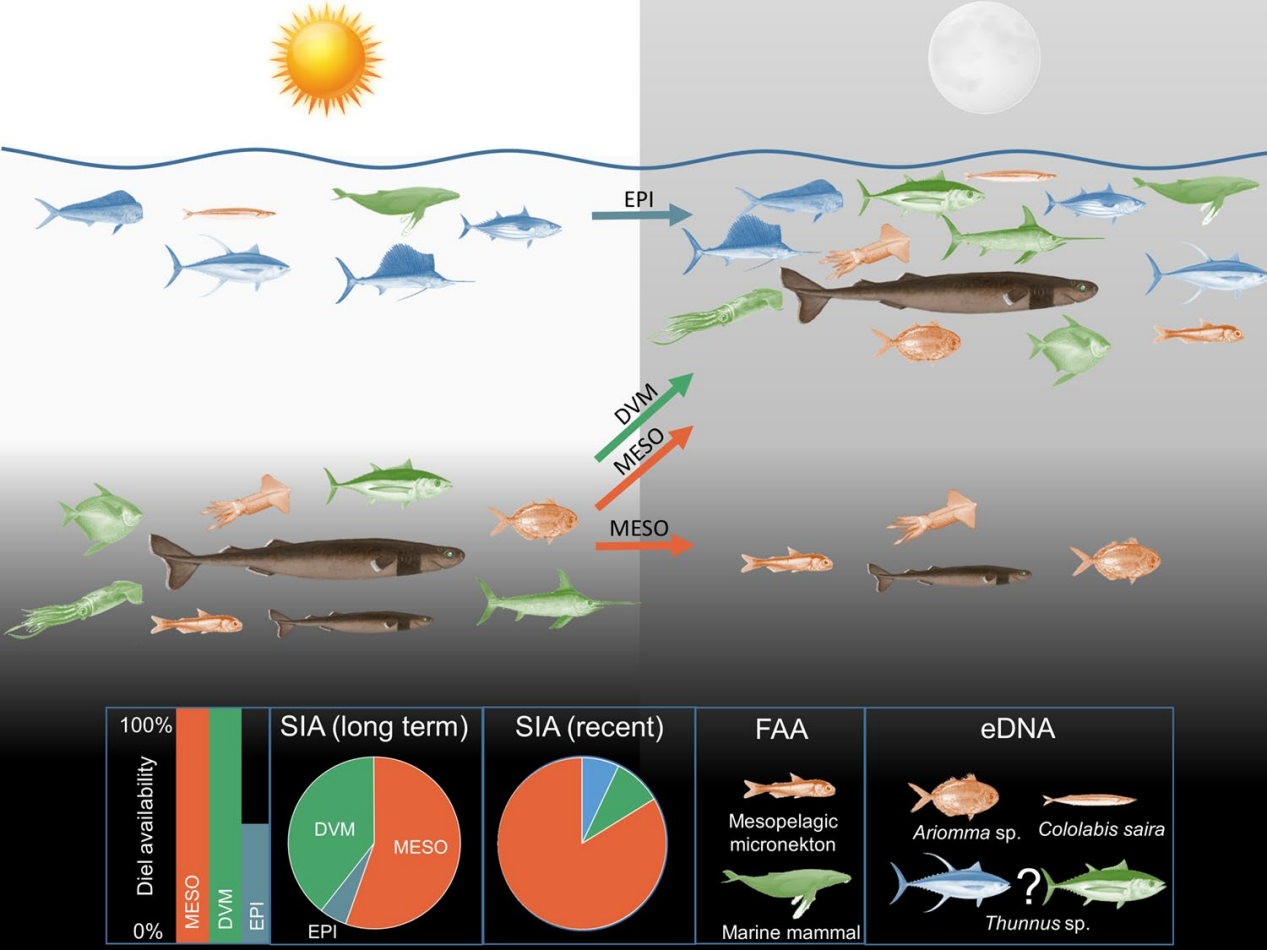
Conceptual figure showing diel shifts in depth distribution of Cookiecutter sharks and different prey groups (DVM, MESO, EPI) as well as relative importance of different prey. The inset shows how the availability of different shark prey groups varies over the course of the diel cycle, as well as information on the diet of Cookiecutter shark revealed by the different chemical tracers. Representative prey of the different prey groups are color coded (MESO: orange, DVM: green, EPI: blue). Note that the question mark by the *Thunnus* sp. in the eDNA inset reflects our inability to assign those stomach contents to either the EPI or MESO prey group.

Using an integrative approach that incorporated multiple biochemical tracers, we gained novel insights into the habitat and trophic ecology of the Cookiecutter shark. Our results indicate that small, micronektonic prey are an important resource for these sharks in the Central Pacific. Surprisingly, our results suggest that large epipelagic prey were less important to the sharks in this study, despite the common belief that they are dominant prey based on bite mark observations. This discrepancy suggests that the high observability of large epipelagic prey to humans may have skewed our understanding of their importance to Cookiecutter sharks. While biochemical tracers generally lack the resolution to identify particular prey species, they were able to identify different functional groups with a high degree of concordance between stable isotope, fatty acid, and eDNA results. Overall, our results indicate that Cookiecutter sharks have a broad diet and have a unique ecological role in pelagic ecosystems, feeding on consumers across all trophic levels, ranging from large, apex predators to small, low trophic level species, in particular species that overlap with the depth distribution of the sharks throughout the diel cycle. We also found evidence of a potential seasonal shift in diet as well as a potential size-based shift in diet and/or habitat, where sharks may start to vertically migrate once they attain a larger size (Fig. 6). This integrative biochemical tracer approach expanded our knowledge of Cookiecutter shark trophic ecology, despite the limited sample size. Only three sharks (20%) had any stomach contents that could be identified using traditional techniques, and those stomach contents were very limited. By using multiple tracers, we were able to use every animal to derive insight into their trophic ecology, increasing our available sample size by 400% in comparison to if we only used traditional stomach content analysis. In addition, because we used different tissues and tracers, we were able to obtain different temporal scales of data that allowed us to make inferences that would not be possible using traditional stomach content analysis without extensive sampling across time, which is generally prohibitively difficult in these ecosystems.

eDNA metabarcoding of gastric fluids was able to detect the presence of prey DNA in shark stomachs, including prey with no identifiable or partially digested tissue material (i.e., *Ariomma*). It is likely that the eDNA results reflect a snapshot of the most recent diet. These results provide new insight into the diet of Cookiecutter sharks, in particular by identifying *Ariomma* and *Cololabis* as prey for this species. Sanger sequencing of partially digested material retrieved from inside two shark stomachs supported the eDNA findings. Our results also demonstrate that eDNA can be extracted and sequenced from stomachs frozen for extended periods of time to accurately identify prey consumed prior to death.

### Importance of different prey groups to Cookiecutter sharks

The trophic ecology of Cookiecutter sharks will be influenced by the degree of spatial and temporal overlap between the sharks and potential prey. Cookiecutter sharks can potentially exploit mesopelagic and vertically migrating species throughout the diel cycle (depending upon the vertical migration of the sharks and the prey), but will only be able to exploit epipelagic species during the night when sharks are near the surface^41^.

Stable isotope, fatty acid, and eDNA results all suggested that Cookiecutter sharks feed more on small prey, whether mesopelagic or epipelagic, than previously thought. Over longer time scales, approximately half their diet is derived from small prey and FAA indicates mesopelagic and deep-sea demersal foraging (Meyer et al. ^11^). Large epipelagic species, though often seen with Cookiecutter shark bite marks in the Central Pacific and across the globe^44,45,54,55^, appear to comprise a relatively small proportion of the overall diet (< 10%) for individuals in this study. However, due to the limited number and geographical extent of samples used in this study, it remains unclear as to whether these patterns will hold in other populations of this globally distributed species, as dietary plasticity is increasingly being acknowledged as a common trait to consumers^56^.

Muscle FAA results indicated that sharks consume large mesopelagic and diel vertically migrating species, small meso- and epipelagic species, and marine mammals, which matches SIA results. These results were also concordant with eDNA results, as four sharks (30%) had *Ariomma* sp. (a small mesopelagic fish) DNA in their stomach, and one shark (8%) had *Cololabis saira* (a small epipelagic fish). *Thunnus* DNA was also found in two sharks (15%), but we were not able to resolve it to species level, so it’s unclear whether it was a species that used mesopelagic (e.g., Bigeye, Albacore) or epipelagic (e.g., Yellowfin, Skipjack) resources. However, it is important to note that primers used in the eDNA study could not identify invertebrates. FAA results indicated the Cookiecutter sharks were most similar to *Somniosus* spp., based on high levels of 18:1ω9 and 16:1ω7, which suggest consumption of marine mammal blubber (^57,58^). *Somniosus* spp. are known to opportunistically feed upon marine mammals^59–61^, further suggesting that marine mammals are prey for larger Cookiecutter sharks. The exceptionally high 18:1 and 16:1 may also come from the skin and subdermal fat from teleost and elasmobranchs (Every et al.^33^) as Cookiecutter sharks remove relatively high proportions of these tissues owing to their unique ectoparasitic hunting. While the FAA suggested that large epipelagic species and cephalopods were the most dissimilar to Cookiecutter sharks, which supports SIA results, the similarity between Cookiecutter and Mako sharks suggests squid, and a mix of epipelagic and mesopelagic teleosts may be important prey items (Preti et al.^62^).

Our finding that large species that undergo diel vertical migration are an important prey resource for Cookiecutter sharks is also consistent with published data. Papastamatiou et al.^44^ reported on the frequency of Cookiecutter shark bites on fishes (n = 15,107 over 51 weekly surveys) at the Honolulu Fish Market, all of which were included as prey in the SIA component of this study [Swordfish, Opah (*Lampris guttatus*), Pomfret (*Brama japonica*), Wahoo (*Acanthocybium solandri*), Sailfish (*Istiophorus platypterus*), Striped Marlin (*Kajikia audax*), Blue Marlin (*Makaira nigricans*), Skipjack (*Katsuwonus pelamis*), Yellowfin Tuna (*Thunnus albacares*), Bigeye Tuna (*Thunnus obesus*)]. Of these species, Swordfish, Opah, Pomfret, and Wahoo clustered in our DVM prey group, while the rest were in our EPI prey group. Swordfish and Opah had the greatest frequencies of bite marks by large margins, with 16.9% (± 15.6 SD) of Swordfish and 13.9% (± 10.5) of Opah having fresh bites and 87.8% (± 25.0) of Swordfish and 33% (± 8.3) of Opah having healed bite scars. All of the other species had relatively low incidences of fresh (1.7–3.4%) and healed (0–6%) bites^44^.

Mixing models are inherently sensitive to the selection of sources and TDFs^63^. We used the approach described by Smith et al.^64^ to investigate how well the TDFs and three sources we used fit our consumer data. We found that our muscle data were well bounded by the mixing polygons, with only one animal falling outside the 95% contour of the mixing polygon, suggesting that our sources and TDFs were generally appropriate. For liver, four animals fell outside the 95% contour of the mixing polygon, suggesting a poorer fit, but one that was still generally suitable. It is possible that we have not accounted for all the potential sources given the diversity of potential prey items and lack of stable isotope data for many potential pelagic prey, in particular mesopelagic species. It appears that we may be missing a source with more depleted δ^13^C than any of the current sources. In addition, while the TDFs appear to be generally suitable, it is unclear how suitable muscle and liver TDFs from a small coastal Carcharhiniform shark will be for a mesopelagic Squaliform shark. However, until controlled feeding studies are done on a species similar to Cookiecutter sharks, we are restricted to using available TDFs. Furthermore, Hussey et al.^65^ found that TDFs narrowed with increasing trophic level, yet how this might impact the Cookiecutter shark, which feeds across all trophic levels, is unclear. While there are important caveats associated with our analyses, the strong degree of concordance between the different approaches provides support for our model selection, analyses, and interpretations.

### Evidence for seasonal dietary changes

Stable isotope mixing model results for liver suggest that recent diet of the sharks was dominated by MESO prey, a result that was consistent using either set of liver TDFs. Because liver represents the diet over the prior weeks to few months prior to death, it suggests small meso- and epi-pelagic prey were the primary prey for Cookiecutter sharks compared to the longer time frame integrated by muscle that includes substantial contributions from DVM prey. This interpretation is supported by FAA. The FA profile of Cookiecutter shark livers, which will reflect more recent feeding (< 3 and up to 12 weeks), is most similar Little Gulper Shark (*Centrophorus uyato*) and the Birdbeak Dogfish (*Deania calcea*), which are both mesopelagic and deep-water species that primarily feed upon mesopelagic micronekton. Interestingly, the low level of PUFAs (22:6ω3 and 20:5ω3) and high ω9 FAs (in this case 44.48 ± 3.64 18:1ω9) observed in shark liver have been used as indicator of essential FA deficiency in young bull sharks^32^, and may indicate poor nutrition. Liver δ^15^N and FAA values suggest sharks recently fed more on small mesopelagic species, which have a lower nutritional value than larger species such as marine mammals and other large diel vertically migrating and epipelagic species^66,67^. If true, it may suggest that this seasonal shift in diet is necessitated by a lack of availability of larger, more energetically rich prey, either as a result of seasonal movements of sharks or their prey. Indeed, Papastamatiou et al.^44^ observed an apparent seasonal shift in frequency of fresh bites on fishes and suggested this may reflect a seasonal shift in the distribution of Cookiecutter sharks. However, this seasonal diet shift to less nutritional prey is speculative and more research is needed to address this hypothesis.

### Shift in trophic ecology with length

We found no significant relationship between the shark length and stable isotope composition but recognize the size range of sharks in this study was limited, particularly for smaller individuals. The variability in δ^15^N values among larger sharks (> 30 cm) may reflect individual variability in diet or habitat use, with different individuals exhibiting differential reliance on mesopelagic vs epipelagic resources. However, the smallest shark had one of the highest δ^15^N and lowest δ^13^C values, which suggests increased use of mesopelagic resources. There is an increase in baseline ecosystem δ^15^N values with increasing depth so the elevated δ^15^N values could indicate increased reliance upon a mesopelagic suspended particulate based food web as opposed to prey directly connected to surface productivity^68–70^. It is possible that smaller individuals feed entirely in the midwater food web without diel vertical migration unlike larger sharks. This result is concordant with FAA that found high levels of 20:5ω3 in the smallest shark, which indicates feeding in deeper, colder waters in deep-sea food webs and on lower trophic level prey (^71–73^; Meyer et al.^11^) including cephalopods (^28,71^). Estimated size at birth is 14–15 cm^74^, so it is possible that the small shark’s stable isotope composition was influenced by maternal contributions^75,76^. Given that this shark was larger (17 cm) than the estimated size of birth and was isotopically distinct from the larger sharks, this interpretation seems less likely. While this result is interesting, it remains speculative due to the small sample size.

While our sample size is limited, SIA and FAA results suggest that diel vertical migration in Cookiecutter sharks may develop as they grow, with small sharks remaining in and feeding in mesopelagic habitats throughout the diel cycle. Alternatively, they may vertically migrate but be restricted to consuming small mesopelagic prey that also vertically migrate. High levels of 18:1ω9, and 18:1ω7 also indicated that large sharks had the highest potential consumption of marine mammal blubber (Waugh et al.^58^) and that sharks < 30.5 cm (n = 5) had significantly different FA profiles than sharks > 30.5 cm (n = 9). We hypothesize that these patterns may indicate that there is an ontogenetic shift in diet or habitat. This interpretation is consistent with Papastamatiou et al.^44^ who found that Cookiecutter shark bites on fishes in the Honolulu Fish Market were almost entirely from sharks estimated to be > 25 cm TL based on measured bite sizes. Papastamatiou et al.^44^ also reported that Swordfish had significantly higher occurrence of bites from larger sharks (> 40 cm TL), which they suggested may reflect a size or sex based shift in foraging ecology, as male sharks are rarely > 40 cm TL^41^. Unfortunately, we did not get eDNA data from sharks < 30 cm, so eDNA results could not provide any insight into this potential length-based shift.

Size-based shifts in diet or habitat are ubiquitous in chondrichthyan fishes, but poorly understood in mid- and deep-water species^77^. Sex and size-based segregation have been noted in other deep-water squaliform sharks, including species in the family Centrophoridae (e.g. *Cetrophorus, Deania*), Somniosidae (e.g. *Scymnodon*), and Etmopteridae (e.g. *Etmopterus, Centroscyllium*)^78–82^. In particular, *Etmopterus princeps* and *Centroscyllium fabricii* are noted to show an ontogenetic shift in habitat, with younger sharks having a deeper distribution than older ones^81^. In addition, some deep-water species may move to deeper waters and give birth^3^, suggesting that Cookiecutter sharks may be born at the deeper part of their depth distribution. While we provide some evidence that Cookiecutter sharks exhibit a length-based shift in habitat and/or diet, our biochemical tracer results do not allow us to further differentiate between these factors. Small sharks could vertically migrate but be constrained to consuming small prey due to gape limitations, though gape limitation seems unlikely given their dentition, or they may indeed remain at depth and feed primarily on mesopelagic micronekton. This size threshold may also reflect change in swimming ability, with larger sharks being able to swim more rapidly to feed upon larger, faster moving prey.

Understanding the trophic ecology of this poorly known predator sheds important insight into its ecological role in epi- and mesopelagic ecosystems. Understanding the ecological role of this globally distributed predator, and other similar mesopelagic predators, is fundamental to understanding food web dynamics in pelagic ecosystems and necessary for effective management. How relevant our findings are to other populations of Cookiecutter sharks around the world will remain unknown until additional studies are conducted. We found that a multi-tracer approach increases our ability to make ecological inferences, especially with small sample sizes, when studying these types of species and food webs.

## Methods

Cookiecutter sharks (n = 15; 9 M, 6 F; mean TL = 33.5 cm ± 6.6 SD; min TL = 17.1 cm, max TL = 43.1 cm, mean mass = 213 g ± 113 SD) were collected off Hawaii in August and September of 2013 by the Monterey Bay Aquarium onboard the NOAA ship R/V Oscar Sette using a Isaacs-Kidd midwater trawl and Cobb trawl fishing primarily at depths between 60 and 150 m, but down to 600 m. While the size at maturity is not well characterized, it has been reported to be ~ 36 cm for males and ~ 39 cm for females^41^. Based on these criteria, three males (43%) and two females (33%) were considered to be mature. Sharks were held in an aquarium on board the vessel until they expired, generally after several hours, at which point they were stored in a -20 °C freezer until they were processed. Sharks were thawed and their stomachs removed for eDNA analysis, and liver samples and muscle samples from the dorsal musculature behind the gills were collected for SIA and FAA. Liver and muscle samples were stored frozen at − 20 °C until they were analyzed. All animals were collected under the auspices of the Monterey Bay Aquarium following the Association of Zoos and Aquariums Accreditation Standards and permits and ethical approvals were obtained from the Monterey Bay Aquarium’s Animal Welfare and Research Oversight Committees. All methods are reported in accordance with ARRIVE guidelines.

Muscle and liver samples were collected because they have different isotopic incorporation rates and as a result integrate dietary information over different periods of time. Muscle has a slower incorporation rate and reflect a shark’s diet integrated over long periods of time, whereas liver, being more metabolically active, has a more rapid incorporation rate and will integrate diet over shorter time frames^20,83,84^. There are no incorporation (turnover) rate studies for mesopelagic sharks or fishes, but we use the allometric relationship from Weidel et al.^85^, which was developed for teleosts but has been reported to be applicable to sharks^20^. The estimated half-life of δ^13^C based on the mass of sharks in this study is an average of ~ 78 days. If true, muscle will reflect the previous 3 – > 9 months (e.g., 50 to > 88% turnover), and liver would be a shorter time frame. Further, lipids are more metabolically active than proteins, such that FAs reflect diet at shorter time frames than stable isotopes (weeks to months) (Beckmann et al.^30^). Muscle FA samples begin to reflect a change in diet within 3 weeks and are fully transformed by 16 weeks (Beckmann et al.^30^). As with isotopic incorporation, liver FAs have a quicker incorporation rate, reflecting diet from within 3–12 weeks (Beckmann et al.^30^), though the rate likely varies across species.

Because urea and lipid content influence the isotopic composition of elasmobranch tissue^10,86,87^, all shark tissues were both lipid and urea extracted following Kim and Koch^87^. Approximately 10 mg of liver was freeze dried, then lipid extracted three times. Each round of lipid extraction consisted of 20 mL 2:1 chloroform:methanol solution, 10 min of ultrasonication, and 24 h on a shaker table. After lipid extraction, liver samples were urea extracted with three rinses in deionized water with 10 min of ultrasonication. Samples were analyzed in triplicate at the Stable Isotope Lab of University of California Merced on a continuous flow isotope ratio mass spectrometer (ThermoScientific Delta V Plus) coupled to an Elemental Analyzer (Costech 4010) with a Conflo IV. Isotopic composition is expressed using standard δ notation, using Vienna Pee Dee Belemnite as the standard for δ^13^C values and atmospheric AIR nitrogen as the standard for δ^15^N values. The liver samples were run with three reference materials (Acetanilide [Costech] and two glutamic acids [USGS 40 and USGS 41a]) and analytical precision for δ^13^C and δ^15^N values was < 0.1‰ and 0.2‰, respectively. TDFs were estimated based on the average difference of captive feeding study shark liver and prey (i.e., δ^13^C_liver_–δ^13^C_prey_) using the previously published isotopic composition of squid and tilapia (Kim et al. ^20,53^). The TDF standard deviation is based on the propagation of error from shark and prey δ^13^C and δ^15^N values.

### Stable isotope analysis (SIA)

Lipid was extracted using a 2:1 chloroform:methanol solution and urea was extracted using deionized water rinses^87^. Samples were then lyophilized and homogenized in a Spex/CertiPrep 5100 mill. Approximately 500 μg of tissue were weighed into tin boats and analyzed at the Stable Isotope Laboratory at UCSC using an Elemental Analyzer (Carlo Erba) coupled to a continuous flow isotope ratio mass spectrometer (Thermo-Finnigan Delta XP). Isotopic composition is expressed using standard δ notation, using Vienna Pee Dee Belemnite as the standard for δ^13^C values and atmospheric AIR nitrogen as the standard for δ^15^N values. Analytical precision, based on an internal lab standard (Pugel), was < 0.2‰ for δ^15^N and < 0.1‰ for δ^13^C values.

The stable isotope composition of potential prey of Cookiecutter sharks around Hawaii was extracted from the literature^68,88–93^ or through sampling fishes (14 species, ~ 10 samples/species) at the Honolulu Fish Market (Appendix S1: Table S1, Fig. S1). We only included potential prey values from the literature that accounted for lipid content through either chemical extraction or mathematical adjustment based on tissue C:N, or did not require lipid extraction due to low lipid content of tissues (See Supplemental Materials for more detail). All potential prey species sampled as part of this study were lipid extracted using a 2:1 chloroform:methanol solution. When there were multiple stable isotope values for a particular prey group, we aggregated them into a single mean and standard deviation (SD) value by resampling 1000 values from each prey δ^13^C and δ^15^N distribution in the group and combining them into a cumulative distribution for that prey group. We grouped potential prey into three groups using k-means clustering. We limited the number of prey groups to three because we were only using two tracers (δ^13^C and δ^15^N), and stable isotope mixing models are most effective when the number of sources used is *n* + 1 (*n* = number of tracers)^63^. These groups generally clustered based on their habitat type. The DVM group (mean ± SD δ^13^C: − 17.9 ± 1.0, δ^15^N: 12.5 ± 0.5) was primarily comprised of large species that exhibit diel vertical migration as well as marine mammals, though there were a few large meso- and epipelagic species in this group as well. The MESO group (δ^13^C: − 17.9 ± 0.9, δ^15^N: 7.3 ± 0.7) was entirely comprised of small mesopelagic and epipelagic micronektonic and forage species. The EPI group (δ^13^C: − 17.1 ± 1.0, δ^15^N: 10.5 ± 0.5) was primarily made of large epipelagic species, though there were some mesopelagic and vertically migrating species in this group as well. We used the Bayesian stable isotope mixing models MixSIAR^94^, using the isotopic composition (δ^13^C, δ^15^N) of the sharks and their potential prey groups (i.e., DVM, MESO, EPI as described above), to estimate the relative importance of different prey groups to sharks over shorter time frames using liver and longer time frames using muscle. The model was run with 300,000 Markov chain Monte Carlo (MCMC) simulations (200,000 burn-in) and showed good convergence based on Gelman-Rubin (all variables < 1.01) and Geweke (no scores outside ± 1.96 in any chain) diagnostics. Uninformative priors were used.

### Fatty acid analysis (FAA)

Muscle and liver samples were freeze-dried prior to transportation to the University of Adelaide Waite lipid laboratory, where samples were analyzed for fatty acid (FA) composition. Total lipid was extracted from samples > 0.12 mg dry weight (Meyer et al.^11^) using a 2:1 chloroform:methanol as per Folch et al.(^95^). Individual FAs were cleaved from the glycerol backbones via transmethylation with 1% H_2_SO_4_ in methanol at 70 °C for 3 h. Resulting FAs were extracted with n-heptane (2 ml) and transferred into gas chromatography (GC) vials. Subsequently, the FAs were separated, identified, and quantified using GC analysis (Hewlett-Packard 6890, CA, USA) with a flame ionisation detector, a split injector, and a BPX-70 capillary column (50 m × 0.32 mm internal diameter) with a 0.25-µm film thickness (SGE, Victoria, Australia). The operating conditions of the GC and fatty acid identification using the GLC 463 external standard (Nu-Chek Prep Inc, MN, USA) are detailed in Kartikasari et al. (^96^). FA results are expressed as a proportion of the total identified compounds and only those with means > 0.1% were included in the subsequent statistical analyses.

We investigated whether Cookiecutter shark muscle and liver FA profiles differed by sex and length, grouping animals into four size classes: extra small (< 29 cm TL, n = 1), small (29–30.5 cm TL, n = 4), medium (30.6–35 cm TL, n = 4), and large (> 35 cm TL, n = 5). We also compared Cookiecutter FA profiles to those from chondrichthyans occupying deep sea (meso- and baythpelagic > 300 m deep), deep demersal (> 300 m deep associated with the benthos), and epipelagic habitats (< 300 m deep) (as in Meyer et al.^11^, sup material) based on Fishbase listings (Froese & Pauly, ^97^) and IUCN Red List assessment’s habitat descriptions (www.iucnredlist.org). Additionally, Cookiecutter shark FA profiles were compared to FA profiles from potential prey items from the Pacific and Southern Ocean (profiles sourced from the literature, metadata and habitat allocations in Supplemental Methods). The majority of the sourced chondrichthyan and prey profiles are the species means as reported in the literature. Prey items were grouped into the same categories as used in the SIA (DVM, EPI, MESO), with the exception of additionally separating marine mammals and cephalopods from the DVM teleosts.

Multivariate statistical analyses in PRIMER7 (with PERMANOVA)^98^ were used to assess if and how FA profiles differed across biological factors (sex and size), habitat type, and prey groups. Permutational analysis of variance (PERMANOVA) tests with Monte Carlo simulations [denoted as p(MC)] were run on Bray–Curtis similarity matrices calculated from the square-root transformed profile data to determine if categorical groups were significantly distinct (p(MC) < 0.05). Where groups were significantly different (determined using PERMANOVAs), similarity percentage analysis (SIMPER) was used on the square root transformed data, to quantify which groups within a factor were most dissimilar, and which FAs were driving pairwise dissimilarities. SIMPER analysis was also used to quantify the dissimilarity between the Cookiecutter sharks and individual shark species. Principal Coordinates Analysis (PCO) was used to visualize the differences between groups, with FA correlations > 0.7 overlaid as vectors on the PCO, indicating direction and magnitude of the correlation.

### Environmental DNA (eDNA)

For each shark (n = 14, one shark’s stomach contents was not examined using eDNA), stomachs were cut open using sterile razor blades. Contents were then squeezed into a sterile weigh boat and homogenized using a razor blade. If prey tissue or bone was present, we sampled the material and extracted separately (n = 4 from 3 sharks). Gastric contents were transferred to a sterile 5 mL falcon tube and vortexed for 3–5 min. Between the treatment of each stomach sample, scissors and tweezers were sterilized with the following steps: wash with 0.05% bleach solution, submerge in 96% ethanol, rinse in RNAse Away and then flame.

#### DNA extraction and PCR amplification

DNA extractions were performed using the DNeasy Blood and Tissue Kit (Qiagen, USA) following the manufacturer’s protocol with minor modifications (see Supplemental Methods). Of the 14 shark stomachs, 11 stomachs had sufficient amounts of starting material to proceed forward. Gastric fluid contents from the 11 stomachs were extracted in triplicate; four samples were taken from visible tissue present in three stomachs and were extracted in duplicate. Samples were randomized prior to extraction. DNA concentrations were determined using the Qubit dsDNA HS Assay (Invitrogen, CA, USA). In addition to the stomach samples, we also included negative controls (i.e., extraction blanks) and positive controls in our study (see Supplemental Methods).

We used a 106-bp fragment of the mitochondrial 12S rRNA gene^99^ to assess the vertebrate diversity present in the samples. Primer sequences were F‐5′ ACTGGGATTAGATACCCC and R‐5′ TAGAACAGGCTCCTCTAG. This primer set has previously been validated in a seawater mesocosm study^100^ and used in other eDNA metabarcoding studies^101–103^. These primers target vertebrates (e.g., fishes and marine mammals) but are less likely to amplify DNA from cartilaginous fishes (e.g., sharks, rays) or sea turtles due to base pair mismatches. The primer set does not amplify invertebrates. Unique tags were added to the 5’ ends of the primers to allow for assignment of sequence reads to the correct sample during bioinformatic processing. Before amplification with the tagged primers, all samples were checked for inhibition (see Supplemental Methods). If deemed inhibited, the sample was removed from further processing (n = 1 shark). PCR reaction chemistry and cycling parameters were the same as Port et al.^102^ (see Supplemental Methods). Each DNA extract was amplified in triplicate, along with no template controls (NTCs) using molecular-biology-grade water in lieu of DNA template for each sample.

Prior to our main study, we designed a blocking primer to inhibit amplification of Cookiecutter shark DNA. We tested the blocker with the 12S primers against DNA extracts from Cookiecutter shark and an in-house fish tissue repository as well as a Cookiecutter shark stomach sample and sequenced the amplicons. While the blocker was effective at inhibiting amplification of Cookiecutter shark DNA, there was differential amplification of some species (data not shown). Due to this primer bias, we did not employ the blocker primer going forward. Note that in these preliminary sequencing runs using Cookiecutter shark stomach samples, we detected Opah, *Lampris guttatus*, as a prey item when the blocker was both included and excluded (Opah was not detected in the main study).

#### Sanger sequencing of partially digested tissue samples

PCR products from tissue samples (n = 4 extracts from 3 tissue samples) were commercially Sanger-sequenced with an ABI 3730 × 1 sequencer (Elim Biopharmaceuticals, Inc., Hayward, CA, USA) in both the forward and reverse direction using the same primers as the initial PCR setup. The resulting sequences were trimmed, edited and aligned using Geneious Pro v. 7.1 (Biomatters, NZ) and then compared against the nonredundant GenBank sequence library using the nucleotide BLAST search engine provided by the National Center for Biotechnology Information (NCBI).

#### Next generation sequencing of eDNA in gastric fluid samples

Tagged PCR products for gastric fluid samples yielding sufficient target DNA (n = 30 across 10 sharks) were pooled in equimolar concentration (20 ng DNA per sample) along with controls (n = 8) to create a single library. Note that of the 14 total sharks, three sharks did not have enough starting material for DNA extraction and amplicons from one shark were not visible on a gel. The library was prepared for sequencing using the KAPA low-throughput library prep kit with real-time library amplification protocol (KAPA Biosystems, USA) and sequenced on an Illumina MiSeq platform (250 bp, paired-end) at the Stanford Functional Genomics facility using a 20% PhiX spike-in control (see Supplemental Methods for more information).

#### Bioinformatics and data analysis

We processed the Illumina sequence reads with banzai, a custom Unix-based script (banzai, https://github.com/jimmyodonnell/banzai), following the approach described in Port et al.^102^. Banzai calls third-party programs^104–106^ to move from raw sequence data to a quality-controlled dataset of sequence counts from operational taxonomic units (OTUs). Sequences were demultiplexed and only retained if the tag added during amplification was present on both the forward and reverse reads to eliminate samples with tag jumping^107^. OTUs were compared to a local nucleotide database containing mitochondrial sequences from NCBI using BLAST+^108^. This database—deposited in the Dryad Digital Repository—totaled 12,689 sequences and included the complete mitochondrial genomes as well partial 12S rRNA gene fragments of bony fishes (Actinopterygii), cartilaginous fishes (Chondrichthyes), true seals (Phocidae), sea lions (Otariidae), whales (Cetacea), marine dolphins (Delphinidae), sea otters (*Enhydra*) and birds (Aves) (sequences downloaded September 2014). We also Sanger sequenced Cookiecutter shark tissue using the 12S primers and deposited this 105 bp sequence in the local database. Default blast parameters were used with a few exceptions (see Supplemental Methods). We removed OTUs classified as non-marine vertebrates (e.g., *Homo sapiens*, *Gallus gallus*) as well as unassigned sequences and reads annotated as chimeras.

To account for uneven sequencing depths across samples, we rarefied each sample (stomachs and positive controls) to 100,000 reads using the “rrarefy” function in the R package vegan^109^ and used the positive and negative controls to create conservative thresholds for filtering for false positives (see Supplemental Methods). Sequence counts were analyzed in terms of presence/absence as well as relative abundance. Given biases associated with PCR amplification (e.g. sequencing, rarefaction, etc.) our analysis was semi-quantitative. Rarefied read counts were binned on an order of magnitude scale (i.e., < 100, < 1000, < 10,000 and < 100,000).

## Supplementary Information

Supplementary Information 1.

## Data Availability

Stable isotope, fatty acid, and eDNA data are all available on Dryad.
