# Supplementary material for: Integrating multiple chemical tracers to elucidate the diet and habitat of Cookiecutter Sharks
Source: Sci Rep. 2021 Jun 3;11:11809. doi: 10.1038/s41598-021-89903-z (PMC8175345; doi:10.1038/s41598-021-89903-z)
Supplement: Supplementary file 1 — Supplementary Information 1. [file 41598_2021_89903_MOESM1_ESM.pdf]

Integrating multiple chemical tracers to elucidate the diet and habitat of Cookiecutter Sharks

Aaron B. Carlisle* – School of Marine Science and Policy, University of Delaware
Elizabeth Andruszkiewicz Allan[1] – Department of Civil and Environmental Engineering, Stanford University
Sora Kim – Department of Life and Environmental Sciences, University of California Merced
Lauren Meyer – Southern Shark Ecology Group, College of Science and Engineering, Flinders University
Jesse Port – Center for Ocean Solutions, Stanford University
Stephen Scherrer – Department of Oceanography, University of Hawaii
John O'Sullivan – Monterey Bay Aquarium

*Corresponding author – carlisle@udel.edu

[1] Current affiliation: School of Marine and Environmental Affairs, University of Washington

Supplemental Materials

Stable Isotope Analysis (SIA)

The effect of lipid content on $\delta^{13}C$ values has been well described in the literature and necessitates the removal of lipid from tissues using chemical extraction methods or accounting for lipid content by mathematically adjusting tissue $\delta^{13}C$ values based on their C:N (Pinnegar and Polunin 1999, Post et al. 2007, Martinez del Rio et al. 2009, Carlisle et al. 2016). The stable isotope values for potential prey from the literature (Table S1) either accounted for lipid content through chemical extraction, mathematical adjustment, or did not need to account for lipid due to the low lipid content of tissues. Lipids were chemically extracted using petroleum ether (Witteveen et al. 2009, Carlisle et al. 2015), hexane (Graham 2007, Graham et al. 2007), or 2:1 chloroform:methanol (Gould et al. 1997). One study used mathematical correction (Choy et al. 2015), while several values for squid did not account for lipid due to the low lipid content of the tissues (Parry 2003, 2008). In addition, we used trophic discrimination factors (TDFs) that were calculated using different lipid extraction methods (petroleum ether, 2:1 chloroform:methanol),

which has the potential to affect the stable isotope composition, and resulting calculated TDFs, of the samples. However this potential source of variability remains undescribed and unquantified. Optimally, all studies would use the same approach to accounting for lipids. However, we are unable to control for lipid extraction methods used in the literature and we contend that it is more important that all the studies somehow account for lipid content rather than use the same method of accounting for lipid content.

Species	ID	Group	$\delta^{13}C$		$\delta^{15}N$		Refs	n
			Mean	SD	Mean	SD		
Albacore tuna (*Thunnus alalunga*)	alb	DVM	-18.7	0.6	12.4	1.2	1	3
Swordfish (*Xiphias gladius*)	swo	DVM	-16.7	0.7	13.0	1.6	1,2	9
Opah (*Lampris guttatus*)	opa	DVM	-18.1	0.7	11.8	1.0	1,2	9
Neon flying squid (*Ommastrephes bartrami*)	nfs	DVM	-19.5	1.2	12.0	1.7	4,5,6	na
Wahoo (*Acanthocybium solandri*)	ono	DVM	-17.2	0.4	12.0	1.4	1	11
Striped marlin (*Kajikia audax*)	str	DVM	-17.3	0.4	12.8	1.0	1	10
Northern right whale dolphin (*Lissodelphis borealis*)	nrw	DVM	-18.7	0.2	11.8	1.9	6	na
Humpback whale (*Megaptera novaeangliae*)	hbw	DVM	-18.0	0.9	13.0	1.4	9	na
Pacific pomfret (*Brama japonica*)	pom	DVM	-18.5	1.0	12.7	2.8	1,6	10
Escolar (*Lepidocybium flavobrunneum*)	esc	DVM	-16.5	0.5	13.0	2.8	1	10
Tropical two-wing flyingfish (*Exocoetus volitans*)	fly	MESO	-18.0	0.7	6.3	3.0	2	na
Miscellaneous forage fish	mff	MESO	-19.7	0.5	7.8	3.5	7	na
Common fangtooth (*Anoplogaster cornuta*)	fan	MESO	-18.7	0.5	7.8	1.2	2	na
Lanternfish (Myctophidae)	myc	MESO	-18.4	1.4	7.3	2.1	2,7	na
Sloane's viperfish (*Chauliodus sloani*)	vip	MESO	-17.1	1.4	8.2	2.9	2	na
Bristlemouth (*Cyclothone* spp.)	bris	MESO	-17.2	2.1	8.1	3.2	2	na
Bobtail eel (*Cyema atrum*)	eel	MESO	-17.3	0.3	7.0	2.0	2	na
Ribbon sawtail fish (*Idiacanthus fasciola*)	rsf	MESO	-17.7	0.9	6.6	1.4	2	na
Pelagic shrimp (Oplophoridae)	shr	MESO	-16.9	0.1	6.6	1.0	3,8	na
Bigeye tuna (*Thunnus obesus*)	bet	EPI	-16.7	0.8	11.7	1.2	1,2,3	9
Purpleback flying squid (*Stenoteuthis oualaniensis*)	pfs	EPI	-18.3	0.6	10.5	2.6	4,5,7	na
Mahi mahi (*Coryphaena hippurus*)	mah	EPI	-16.4	1.1	10.7	1.7	1	9
Sailfish (*Istiophorus platypterus*)	sai	EPI	-17.2	0.5	11.2	0.7	1	5
Skipjack (*Katsuwonus pelamis*)	skj	EPI	-16.9	0.9	9.9	1.9	1,2	12
Blue marlin (*Makaira nigricans*)	blm	EPI	-16.3	1.2	11.5	1.8	1,2	9
Spearfish (*Tetrapturus angustirostris*)	spr	EPI	-17.0	0.7	11.3	2.1	1	8
Yellowfin tuna (*Thunnus albacares*)	yft	EPI	-16.6	1.0	10.0	1.8	1,2,8	9
Long-snouted lancetfish (*Alepisaurus ferox*)	lan	EPI	-18.4	1.7	9.7	1.9	2	na
Snake mackerel (*Gempylus serpens*)	snak	EPI	-17.0	0.7	10.0	1.4	2	na
Sawtooth eel (*Serrivomer sector*)	saw	EPI	-17.1	1.9	9.2	1.7	2	na

Table S1: Stable isotope composition of potential Cookiecutter Shark prey from around Hawaii and the Central Pacific. Data were extracted from the literature or are from this study. Studies accounted for lipid content through chemical extraction or mathematical correction, or did not need to account for lipid due to the low lipid content of tissues. References are 1 (this study), 2 (Choy et al. 2015), 3 (Graham 2007), 4 (Parry 2003), 5 (Parry 2008), 6 (Gould et al. 1997), 7 (Carlisle et al. 2015), 8 (Graham et al. 2007), 9 (Witteveen et al. 2009).

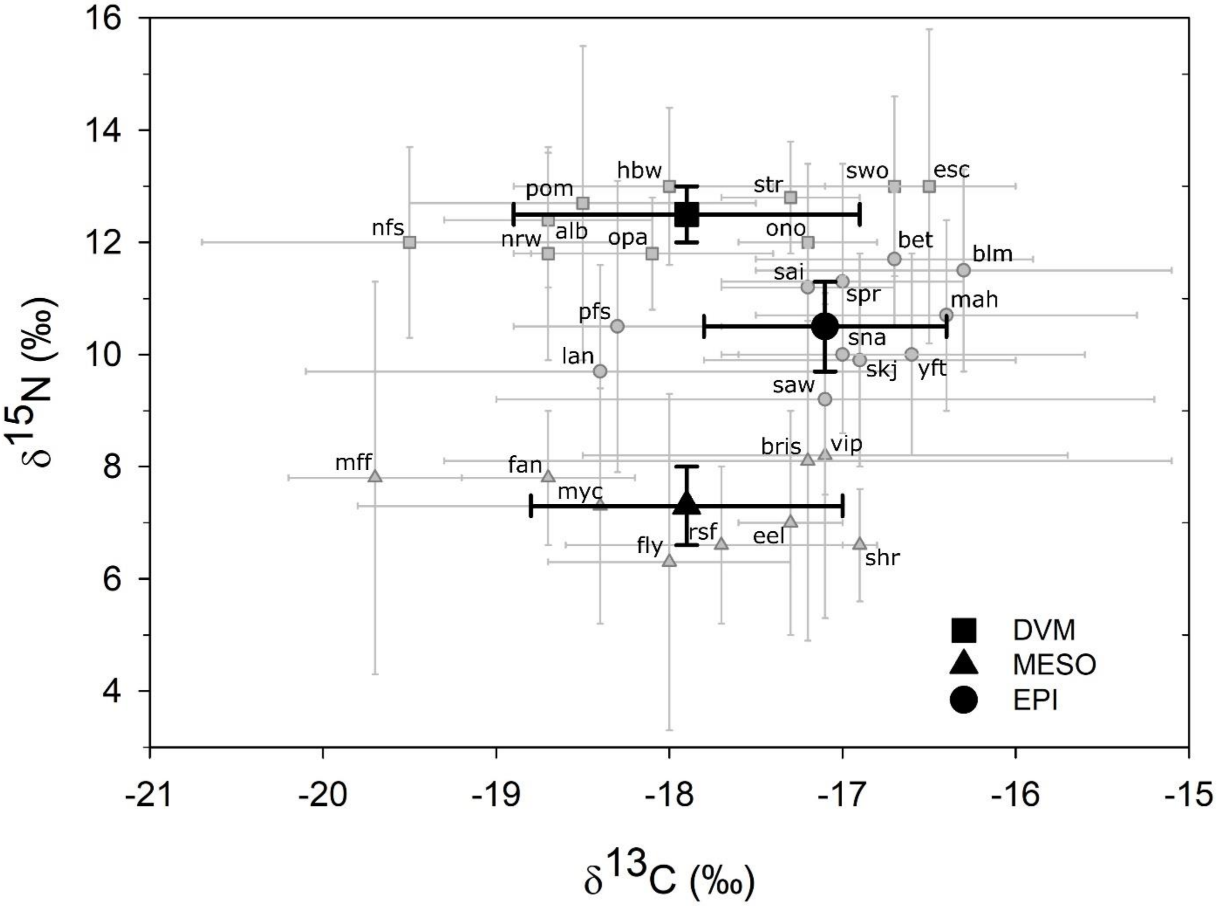

Figure S1: Potential prey field for Cookiecutter Sharks around Hawaii. Mean (SD) δ^{13}C and δ^{15}N of the three primary groups (DVM, MESO, EPI) are shown in black, whereas the mean (SD) δ^{13}C and δ^{15}N of constituent species are shown in grey. Prey species are: alb (Albacore Tuna), bet (Bigeye Tuna), blm (Blue Marlin), bris (Bristlemouth), eel (Bobtail Eel), esc (Escolar), fan (Common Fangtooth), fly (Tropical Two-Wing Flyingfish), hbw (HumpbackWhale), lan (Long-Snouted Lancetfish), mah (Mahi Mahi), mff (miscellaneous forage fish), myc (Lanternfishes), nfs (Neon Flying Squid), nrw (Northern Right Whale Dolphin), ono (Wahoo), opa (Opah), pfs (Purpleback Flying Squid), pom (Pacific Pomfret), rsf (Ribbon Sawtail Fish), sai (Sailfish), saw (Sawtooth Eel), shr (Pelagic Shrimp), skj (Skipjack), sna (Snake Mackerel), spr (Spearfish), str (Striped Marlin), swo (Swordfish), vip (Sloane’s Viperfish), yft (Yellowfin Tuna).

Fatty Acid Analysis (FAA)

Species/ habitat	Cookiecutter shark	Deep sea	Deep sea – demersal	Pelagic
n	14	20	15	8
14:0	1.15 ±0.33	0.51±0.35	0.59 ±0.53	1.58 ±1.31
16:0	19.61 ±1.40	15.68±3.48	19.73 ±1.84	20.06 ±3.80
18:0	3.46 ±0.48	5.96±2.07	7.32 ±3.50	11.79 ±3.05
20:0	0.56 ±0.86	0.11±0.02	0.06 ±0.02	0.11 ±0.11
∑SFA	26.02 ±3.90	22.19±4.73	27.72 ±4.82	33.68 ±5.19
16:1ω7	7.50 ±1.30	1.91 ±1.77	1.65 ±2.17	2.28 ±0.99
18:1ω9	44.48 ±3.64	16.37 ±4.89	11.72 ±1.49	12.29 ±4.91
18:1ω7	4.60 ±0.45	4.41 ±1.96	5.35 ±2.43	6.49 ±2.39
20:1ω9	3.99 ±0.82	3.83 ±4.21	2.91 ±1.56	2.71 ±1.15
22:1ω9	0.68 ±0.49	0.75 ±0.51	0.72 ±0.36	0.89 ±0.62
∑MUFA	61.25 ±6.07	22.87 ±11.65	17.08 ±9.03	18.27 ±11.97
18:2ω6	1.46 ±0.76	0.84 ±0.31	1.02 ±0.85	0.49 ±0.35
20:4ω6	1.63 ±0.35	4.57 ±1.59	5.11 ±2.42	5.35 ±2.51
20:5ω3	1.05 ±0.88	3.14 ±1.61	5.81 ±3.12	2.21 ±1.13
20:3ω6	0.26 ±0.28	0.19 ±0.20	0.12 ±0.10	0.18 ±0.04
20:2ω6	0.22 ±0.11	0.60 ±0.60	0.43 ±0.48	0.28 ±0.10
22:6ω3	3.36 ±0.71	28.99 ±9.44	28.81 ±6.80	19.18 ±6.47
22:4ω6	0.46 ±0.43	1.30 ±0.81	1.09 ±0.70	2.50 ±1.48
22:5ω3	1.96 ±2.06	3.53 ±1.09	2.59 ±1.27	3.93 ±1.61
∑PUFA	10.66 ±5.78	42.42 ±9.66	44.45 ±6.28	34.07 ±6.79
∑Other	2.07 ±1.23	12.52±6.01	10.75 ±4.65	13.99 ±9.18

Table S2: Chondrichthyan muscle fatty acid (mean % contribution ± SD) profiles. SFA: saturated fatty acids, MUFA: monounsaturated fatty acids, PUFA: polyunsaturated fatty acids.

Species/ habitat	Cookie cutter shark	Deep sea	Deep sea –demersal	Pelagic
n	13	20	13	12
14:0	1.45 ±0.48	1.16 ±1.64	1.16 ±0.27	2.95 ±1.12
15:0	0.59 ±0.11	0.30 ±0.19	0.33 ±0.15	0.52 ±0.22
16:0	20.87 ±1.86	11.42 ±4.50	15.71 ±4.77	16.72 ±3.25
18:0	3.25 ±0.64	2.58 ±1.11	3.52 ±1.00	5.21 ±1.85
20:0	0.32 ±0.07	0.50 ±0.26	0.44 ±0.29	0.14 ±0.04
22:0	0.35 ±0.13	0.05 ±0.03	NR	0.10 ±0.03
24:0	0.29 ±0.21		NR	0.05 ±0.01
**∑SFA**	**27.13 ±2.68**	**15.90 ±5.38**	**21.16 ±5.04**	**25.58 ±5.64**
16:1ω7	7.05 ±1.77	2.58 ±1.46	2.94 ±1.39	6.54 ±4.09
18:1ω9	45.17 ±2.12	27.97 ±5.10	27.06 ±3.87	16.84 ±3.04
18:1ω7	4.41 ±0.93	3.75 ±1.62	3.31 ±0.92	4.79 ±2.07
20:1ω9	4.78 ±1.46	15.89 ±4.78	12.29 ±6.15	4.63 ±3.31
22:1ω9	1.13 ±0.40	3.88 ±1.11	2.34 ±1.14	0.79 ±0.47
**∑MUFA**	**62.55 ±2.89**	**51.84 ±7.96**	**47.94 ±5.01**	**33.59 ±6.32**
18:2ω6	0.86 ±0.18	0.49 ±0.31	0.65 ±0.27	1.08 ±0.36
18:3ω3	0.15 ±0.06	1.24 ±0.17	NR	0.52 ±0.31
20:4ω6	0.60 ±0.15	0.54 ±0.38	1.57 ±1.10	1.47 ±0.60
20:5ω3	0.57 ±0.35	0.80 ±0.59	1.47 ±1.30	4.96 ±3.00
20:3ω6	0.14 ±0.12	0.35 ±0.03	NR	0.14 ±0.04
20:2ω6	0.28 ±0.07	0.26 ±0.17	0.33 ±0.18	0.32 ±0.08
22:6ω3	3.29 ±1.43	3.36 ±3.92	5.33 ±4.26	17.34 ±7.50
22:4ω6	0.16 ±0.10	0.21 ±0.23	0.15 ±0.25	0.73 ±0.42
22:5ω3	1.71 ±0.52	0.60 ±0.39	1.05 ±0.24	3.17 ±0.92
**∑PUFA**	**7.76 ±2.14**	**6.24 ±4.45**	**10.56 ±5.79**	**29.67 ±9.67**
∑Other	2.56 ±1.11	26.02 ±9.40	20.35 ±7.47	11.15 ±1.56

Table S3: Chondrichthyan liver fatty acid (mean % contribution ± SD) profiles. SFA: saturated fatty acids, MUFA: monounsaturated fatty acids, PUFA: polyunsaturated fatty acids.

Fatty acid	New Zealand lanternshark	Pacific sleeper shark	Portuguese dogfish	Black dogfish	Leafscale gulper shark	Greenland shark	Longnose velvet dogfish -ad	Longnose velvet dogfish -jv	Portuguese dogfish (AUS)	Plunket shark	Birdbeak dogfish	Kitefin shark	Southern chimaera	Southern dogfish	Piked dogfish	Spiny dogfish	Greeneye spurdog	Draughtboard shark	Piked Dogfish (BS AUS)	Elephantfish
	**Chondrichthyan common name**																			
14:0	0.30	1.79	0.71	0.38	0.57		0.21	0.57	0.56	0.49	0.57	0.34	0.50	0.40	0.37	0.50	0.31	0.70	0.00	0.36
16:0	14.61	12.81	11.45	12.49	11.99	10.10	16.84	11.76	14.33	19.33	14.50	17.61	19.40	20.40	18.51	13.60	13.70	17.50	22.40	20.36
18:0	4.84	2.27	3.10	4.90	7.69	1.30	6.82	7.56	5.59	5.76	7.73	6.88	4.00	9.34	7.63	5.01	6.65	9.00	6.10	6.95
**∑SFA**	**19.75**	**17.27**	**15.26**	**17.90**	**20.25**	**11.40**	**23.86**	**19.89**	**20.48**	**25.58**	**22.80**	**24.83**	**23.90**	**30.13**	**26.50**	**19.11**	**20.66**	**27.20**	**28.50**	**28.45**
16:1ω7	0.49	5.45				6.70	1.34	0.44	0.70	0.88	1.36	2.07	0.90	1.70	1.21	2.70	0.56			2.22
18:1ω9	20.46	24.34	22.53	20.52	19.12	19.80	13.93	10.74	22.02	12.80	11.69	19.53	8.50	12.20	12.99	19.30	15.73			8.46
18:1ω7	2.97	5.45				7.10	3.71	3.68	1.22	3.58	3.50	4.29	10.10	4.30	3.61	4.61	3.32			4.63
20:1ω9	1.27	5.79				17.90	2.92	2.70	1.60	2.47	3.22	2.44	1.20	1.80	2.88	8.50	2.08			0.71
22:1ω9	0.34	1.38				2.00	0.72	1.16	0.30	0.38	0.31	0.80	0.20	0.50	0.80	1.24	0.32			
**∑MUFA**	**25.54**	**42.41**	**22.53**	**20.52**	**19.12**	**53.50**	**22.62**	**18.72**	**25.84**	**20.11**	**20.08**	**29.13**	**20.90**	**20.50**	**21.49**	**36.35**	**22.02**			**16.02**
18:2ω6	1.04	1.40	0.99	1.15	0.80	1.10	0.55	0.41	0.83	1.16	1.03	0.83	0.90	0.54	1.23	0.61	0.56	0.80	0.10	0.67
20:4ω6	4.20	1.37	5.88	6.97	6.24	1.60	4.36	4.66	4.50	3.64	3.90	6.48	7.58	4.61	3.63	3.30	3.68	5.60	4.60	
20:5ω3	1.89	6.98	3.00	4.73	2.51	5.60	2.29	2.62	3.18	0.86	1.76	2.03	3.40	4.81	1.20	4.91	1.58	4.20	2.10	
20:3ω6	0.07	0.14					0.14	0.00	0.15	0.00	0.13	0.21	0.14	0.12	0.10	0.76	0.16			0.51
20:2ω6	2.14	0.54	0.18	0.19	0.21		0.33	0.00	1.09	0.11	0.50	0.20	1.80	0.31	0.40	0.65	0.28			1.29
22:6ω3	29.82	9.64	33.15	38.67	39.67	8.80	32.15	37.47	27.06	35.29	33.71	24.52	23.20	27.10	32.33	15.95	36.75	23.30	45.80	25.31
22:4ω6	1.09						1.26	1.97	0.74	1.17	2.17	0.92	3.80	0.80	1.17	0.51	1.24	1.00	0.40	1.28
22:5ω3	2.16	2.23	4.11	3.62	4.00	1.70	3.37	6.17	2.98	2.97	4.40	1.94	2.70	3.56	3.49	3.47	3.81	5.30	3.90	4.76
**∑PUFA**	**42.40**	**22.68**	**47.56**	**55.53**	**53.65**	**20.30**	**44.45**	**53.31**	**40.53**	**45.21**	**47.59**	**37.13**	**43.52**	**41.84**	**43.54**	**30.18**	**48.06**	**40.20**	**56.90**	**33.83**
Total % contribution	87.70	82.36	85.35	93.95	93.02	85.20	90.93	91.92	86.84	90.90	90.47	91.09	88.32	92.48	91.54	85.63	90.74	67.40	85.40	78.29

Table S4: Deep-sea chondrichthyan muscle fatty acid (mean % contribution) profiles sourced from the literature. SFA: saturated fatty acids, MUFA: monounsaturated fatty acids, PUFA: polyunsaturated fatty acids.

Fatty acid	Chondrichthyan common name														
	Arctic skate	Roughskin dogfish	Australian sawtail catshark	South China catshark	Sandy-backed stingaree	Long-snouted Skate	Ogilby's ghost Shark	Piked dogfish (FRDC AUS)	Skates	Eaton's skate (KER)	Kerguelen Sandpaper [illegible]	Murray's skate	McCain's skate (ROS)	Eaton's skate (ROS)	Smooth skate
14:0		0.42	0.42	0.46	0.00	0.00	0.00	0.14	0.36	0.74	1.71	0.78	0.80	0.70	1.66
16:0	18.10	16.30	19.74	21.12	20.20	23.00	19.20	20.11	21.20	19.78	22.85	20.06	18.80	18.80	16.65
18:0	4.40	6.73	6.20	4.45	10.50	18.70	6.40	8.31	8.68	5.94	8.50	5.83	5.10	5.80	4.23
**∑SFA**	**22.50**	**23.45**	**26.35**	**26.03**	**30.70**	**41.70**	**25.60**	**28.73**	**30.84**	**26.69**	**33.71**	**26.95**	**24.70**	**25.30**	**22.54**
16:1ω7	3.00	1.39	1.52	1.01				0.72	1.24	0.00	0.00	0.00			7.60
18:1ω9	8.10	11.60	13.20	10.56				11.69	11.24	11.35	13.77	13.41	12.60	12.50	10.68
18:1ω7	6.30	3.31	1.16	3.48				3.15	3.09	5.82	5.75	6.73	9.00	9.40	6.99
20:1ω9	4.40	2.53	2.61	6.93						2.68	1.85	2.80	2.10	2.10	1.08
22:1ω9	0.50	0.59	0.38	0.53						0.96	1.28	1.24			0.30
**∑MUFA**	**22.30**	**19.41**	**18.87**	**22.50**				**15.56**	**15.57**	**20.82**	**22.65**	**24.18**	**23.70**	**24.00**	**26.65**
18:2ω6	1.50	0.71	0.64	0.35	0.30	0.50	0.20	0.34	0.88	0.93	0.43	1.07	3.10	2.80	1.50
20:4ω6	3.30	4.33	4.18	3.37	12.60	7.60	4.10	4.81	7.80	3.83	4.27	3.31	4.90	5.10	3.09
20:5ω3	10.30	1.76	1.90	2.86	3.10	4.80	4.90	1.69	4.81	9.19	5.79	7.03	9.90	9.10	10.02
20:3ω6		0.00	0.19	0.35				0.07	0.10	0.15	0.03	0.07			
20:2ω6		0.27	1.68	0.43				0.21	0.25	0.17	0.20	0.19			
22:6ω3	30.50	32.79	32.28	28.91	27.90	34.80	41.80	41.04	26.54	25.28	15.82	23.33	24.40	25.90	20.87
22:4ω6		0.93	1.36	2.30	2.10	1.40	0.70			0.41	0.82	0.37	1.60	0.00	
22:5ω3	2.20	3.44	3.26	5.91	1.50	2.80	4.20	2.08	3.77	1.99	1.55	1.88	1.10	1.40	1.75
**∑PUFA**	**48.10**	**44.23**	**45.50**	**44.46**	**47.50**	**51.90**	**55.90**	**50.24**	**44.15**	**41.95**	**29.07**	**37.24**	**45.00**	**44.30**	**37.23**
Total % contribution	92.90	87.09	90.73	92.99	78.20	93.60	81.50	94.53	90.56	89.45	85.43	88.37	93.40	93.60	86.42

Table S5: Deep-sea demersal chondrichthyan muscle fatty acid (mean % contribution) profiles sourced from the literature. SFA: saturated fatty acids, MUFA: monounsaturated fatty acids, PUFA: polyunsaturated fatty acids

	Chondrichthyan common name							
**Fatty acid**	Shortfin Mako	Porbeagle	Mako (AUS)	Thresher shark ((AUS)	White shark (AUS)	Basking shark	White shark (SA)	Mako (SA)
14:0	2.10	1.40	0.83	0.53	0.81	0.79	4.83	1.32
15:0			0.13	0.15	0.22	0.15		
16:0	17.00	22.00	23.38	15.70	18.55	17.83	18.13	27.88
18:0	6.80	6.70	13.34	14.13	13.79	12.99	11.55	15.02
20:0			0.23	0.26		0.06	0.00	0.00
22:0			0.23	0.30		0.05		
24:0			0.12	0.11		0.00		
**∑SFA**	**25.90**	**30.10**	**38.26**	**31.18**	**33.37**	**31.87**	**34.51**	**44.22**
16:1ω7	2.80	2.50	3.97	1.10	2.13	1.15		
18:1ω9	22.00	12.00	12.14	5.56	11.90	10.13		
18:1ω7	5.10	4.60	11.70	5.94	6.25	5.34		
20:1ω9	4.60	2.80	2.04	1.20	1.94	3.70		
22:1ω9			0.53	0.70	1.94	0.37		
**∑MUFA**	**34.50**	**21.90**	**30.38**	**14.50**	**24.16**	**20.69**		
18:2ω6	1.00	0.60	0.00	0.00		0.74	0.71	0.40
20:4ω6	3.00	4.70	4.42	6.16	9.22	1.42	8.88	5.00
20:5ω3	2.60	2.90	0.45	1.84	1.63	3.53	3.83	0.89
20:3ω6			0.22	0.13		0.18		
20:2ω6			0.31	0.38		0.15		
22:6ω3	21.00	27.00	11.12	28.26	15.52	24.13	15.81	10.62
22:4ω6	1.30	1.20	4.13	1.97	2.44	0.71	2.89	5.33
22:5ω3	3.90	4.50	3.01	4.91	2.75	7.13	3.94	1.30
**∑PUFA**	**32.80**	**40.90**	**23.66**	**43.65**	**31.56**	**37.99**	**37.31**	**24.66**
Total % contribution	93.20	92.90	92.30	89.33	89.09	90.55	71.81	68.88

Table S6: Pelagic chondrichthyan muscle fatty acid (mean % contribution) profiles sourced from the literature. SFA: saturated fatty acids, MUFA: monounsaturated fatty acids, PUFA: polyunsaturated fatty acids. AUS: Australia, SA: South Africa

**Common name (location)**	***Scientific name***	**Habitat**	**Citation**
White shark (AUS)	*Carcharodon carcharias*	pelagic	Pethybridge, Parrish, Bruce, Young, & Nichols, 2014
Basking shark	*Cetorhinus maximus*	pelagic	Pethybridge, Parrish, Bruce, Young, & Nichols, 2014
Portuguese dogfish	*Centroscymnus coelolepis*	deep sea	Økland, Stoknes, Remme, Kjerstad, & Synnes, 2005
Black dogfish	*Centroscyllium fabricii*	deep sea	Økland, Stoknes, Remme, Kjerstad, & Synnes, 2005
Leafscale gulper shark	*Centrophorus squamosus*	deep sea	Økland, Stoknes, Remme, Kjerstad, & Synnes, 2005
Shortfin mako	*Isurus oxyrinchus*	pelagic	Davidson, Sidell, Rhodes, & Cliff, 2011
White shark (SA)	*Carcharodon carcharias*	pelagic	Davidson, Sidell, Rhodes, & Cliff, 2011
Greenland shark	*Somniosus microcephalus*	deep sea	McMeans, Arts, & Fisk, 2012
Arctic skate	*Amblyraja hyperborea*	deep sea - D	McMeans, Arts, & Fisk, 2012
Pacific sleeper shark	*Somniosus pacificus*	deep sea	McMeans, Arts, & Fisk, 2012
New Zealand lanternshark	*Etmopterus baxteri*	deep sea	Pethybridge, Daley, Virtue, & Nichols, 2010
Longnose velvet dogfish -ad	*Centroselachus crepidater*	deep sea	Pethybridge, Daley, Virtue, & Nichols, 2010
Longnose velvet dogfish - jv	*Centroselachus crepidater*	deep sea	Pethybridge, Daley, Virtue, & Nichols, 2010
Portuguese dogfish (AUS)	*Centroscymnus coelopsis*	deep sea	Pethybridge, Daley, Virtue, & Nichols, 2010
Roughskin dogfish	*Centroscymnus owstoni*	deep sea - D	Pethybridge, Daley, Virtue, & Nichols, 2010
Plunket shark	*Proscymnodon plunketi*	deep sea	Pethybridge, Daley, Virtue, & Nichols, 2010
Australian sawtail catshark	*Figaro boardmani*	deep sea - D	Pethybridge, Daley, Virtue, & Nichols, 2010
Birdbeak dogfish	*Deania calcea*	deep sea	Pethybridge, Daley, Virtue, & Nichols, 2010
Kitefin shark	*Dalatias licha*	deep sea	Pethybridge, Daley, Virtue, & Nichols, 2010
Southern chimaera	*Chimaera fulva*	deep sea	Pethybridge, Daley, Virtue, & Nichols, 2010
South China catshark	*Apristurus sinensis*	deep sea - D	Pethybridge, Daley, Virtue, & Nichols, 2010
Southern dogfish	*Centrophorus zeehaani*	deep sea	Pethybridge, Daley, Virtue, & Nichols, 2010
Piked dogfish (TAS AUS)	*Squalus megalops*	deep sea	Pethybridge, Daley, Virtue, & Nichols, 2010
Spiny dogfish (TAS AUS)	*Squalus acanthias*	deep sea	Pethybridge, Daley, Virtue, & Nichols, 2010
Greeneye spurdog	*Squalus chloroculus*	deep sea	Pethybridge, Daley, Virtue, & Nichols, 2010
Sandy-backed stingaree	*Urolophus bucculentus*	deep sea - D	Dunstan, Sinclair, O’Dea, & Naughton, 1988
Long-snouted Skate	*Zearaja nasutus*	deep sea - D	Dunstan, Sinclair, O’Dea, & Naughton, 1988
Draughtboard shark	*Cephaloscyllium isabellum*	deep sea	Dunstan, Sinclair, O’Dea, & Naughton, 1988
Piked Dogfish (BS AUS)	*Squalus megalops*	deep sea	Dunstan, Sinclair, O’Dea, & Naughton, 1988
Ogilby's ghost Shark	*Hydrolagus ogilbyi*	deep sea - D	Dunstan, Sinclair, O’Dea, & Naughton, 1988
Piked dogfish (FRDC AUS)	*Squalus megalops*	deep sea - D	Nichols, Mooney, & Elliott, 2002
Skate	*Raja sp.*	deep sea - D	Nichols, Mooney, & Elliott, 2002

Eaton's skate (KER)	*Bathyraja eatonii*	deep sea - D	Meyer unp. data
Kerguelen Sandpaper Skate	*Bathyraja irrasa*	deep sea - D	Meyer unp. data
Murray's skate	*Bathyraja murrayi*	deep sea - D	Meyer unp. data
McCain's skate (ROS)	*Bathyraja maccaini*	deep sea - D	Jo et al., 2013
Eaton's skate (ROS)	*Bathyraja eatonii*	deep sea - D	Jo et al., 2013
Smooth skate	*Raja senta*	deep sea - D	Budge, Iverson, Bowen, & Ackman, 2002
Elephant fish	Callorhinchus milii	deep sea	Nichols, Mooney, & Elliott, 2002
Shortfin mako	*Isurus oxyrinchus*	pelagic	Vlieg, Murray, & Body, 1993
Poorbeagle	*Lamna nasus*	pelagic	Vlieg, Murray, & Body, 1993

Table S7: Metadata and habitat for chondrichthyan muscle fatty acid profiles. All profiles listed are the published means for the species, location or season. –D indicates demersal habitat use. See literature cited in Supplemental Materials for references.

Common name	Scientific name	*n*	Habitat	Citation
White shark	*Carcharodon carcharias*	7	pelagic	Pethybridge, Parrish, Bruce, Young, & Nichols, 2014
Catshark spp.	*Apristurus sinensis*	Published mean	deep sea -D	Pethybridge, Daley, Virtue, & Nichols, 2010
Portuguese dogfish	*Centroscymnus coelolepis*	5	deep sea	Pethybridge, Daley, Virtue, & Nichols, 2010
Kitefin shark	*Dalatias licha*	Published mean	deep sea	Pethybridge, Daley, Virtue, & Nichols, 2010
Plunket shark	*Proscymnodon plunketi*	3	deep sea	Pethybridge, Daley, Virtue, & Nichols, 2010
Roughskin dogfish	*Centroscymnus owstoni*	6	deep sea -D	Pethybridge, Daley, Virtue, & Nichols, 2010
Chimaera	*Chimaeridae*	Published mean	deep sea	Pethybridge, Daley, Virtue, & Nichols, 2010
Chimaera	*Chimaeridae*	Published mean	deep sea	Pethybridge, Daley, Virtue, & Nichols, 2010
Birdbeak dogfish	*Deania calcea*	3	deep sea	Pethybridge, Daley, Virtue, & Nichols, 2010
New Zealand lanternshark	*Etmopterus baxteri*	6	deep sea	Pethybridge, Daley, Virtue, & Nichols, 2010
Pacific spookfish	*Rhinochimaera pacifica*	3	deep sea	Pethybridge, Daley, Virtue, & Nichols, 2010
Broadnose sevengill shark	*Notorynchus cepedianus*	Published mean	deep sea	Pethybridge, Daley, Virtue, & Nichols, 2010
Shortnose spurdog	*Squalus megalops*	Published mean	deep sea -D	Pethybridge, Daley, Virtue, & Nichols, 2010
Spiny dogfish	*Squalus acanthias*	Published mean	deep sea	Pethybridge, Daley, Virtue, & Nichols, 2010
Shortspine spurdog	*Squalus chloroculus*	Published mean	deep sea -D	Pethybridge, Daley, Virtue, & Nichols, 2010
Shortfin mako (spain)	*Isurus oxyrinchus*	Published mean	pelagic	Guil-Guerrero, Venegas-Venegas, Rincón-Cervera, & Suárez, 2011
Salmon shark (M, summer)	*Lamna ditropis*	Published mean	pelagic	Jayasinghe, Gotoh, & Wada, 2003
Salmon shark (M, winter)	*Lamna ditropis*	Published mean	pelagic	Jayasinghe, Gotoh, & Wada, 2003
Salmon shark (F, summer)	*Lamna ditropis*	Published mean	pelagic	Jayasinghe, Gotoh, & Wada, 2003
Salmon shark (F, winter)	*Lamna ditropis*	Published mean	pelagic	Jayasinghe, Gotoh, & Wada, 2003

Portuguese dogfish	*Centroscymnus coelolepis*	Published mean	deep sea	Remme et al., 2006
Leafscale gulper shark	*Centrophorus squamosus*	Published mean	deep sea	Remme et al., 2006
Black dogfish	*Centroscyllium fabricii*	Published mean	deep sea	Remme et al., 2006
Greenland shark	*Somniosus microcephalus*	Published mean	deep sea	McMeans et al., 2012
Pacific sleepershark	*Somniosus pacificus*	Published mean	deep sea	Schaufler, Heintz, Sigler, & Hulbert, 2005

Table S8: Metadata and habitat for chondrichthyan liver fatty acid profiles. –D indicates demersal habitat use. See literature cited in Supplemental Materials for references.

Common name	Species name	Habitat/taxa	Citation
Glass squid	*Galiteuthis glacialis*	Cephalopod	Field, Nichols, Bradshaw, & Hindell, 2009
Southern Ocean squid	*Histioteuthis eltaninae*	Cephalopod	Field, Nichols, Bradshaw, & Hindell, 2009
Whip-lash squid	*Mastigoteuthis sp.*	Cephalopod	Field, Nichols, Bradshaw, & Hindell, 2009
Greater hooked squid	*Onykia ingens*	Cephalopod	Field, Nichols, Bradshaw, & Hindell, 2009
Neon flying squid	*Ommastrephes bartramii*	Cephalopod	Piché, Iverson, Parrish, & Dollar, 2010
Swordfish	*Xiphias gladius*	DMV/Meso Big	Meynier et al., 2008
Albacore tuna	*Thunnus alalunga*	DMV/Meso Big	Vlieg et al., 1993
Albacore tuna	*Thunnus alalunga*	DMV/Meso Big	Wheeler & Morrissey, 2003
Albacore tuna	*Thunnus alalunga*	DMV/Meso Big	Wheeler & Morrissey, 2003
Bigeye tuna	*Thunnus obesus*	DMV/Meso Big	Peng, Chen, Shi, & Wang, 2013
Escolar	*Lepidocybium flavobrunneum*	DMV/Meso Big	Nichols et al., 2002
Slender tuna	*Allothunnus fallai*	DMV/Meso Big	Nichols et al., 2002
Moonfish	*Lampris guttatus*	DMV/Meso Big	Nichols et al., 2002
Southern bluefin tuna	*Thunnus maccoyii*	EPI Big	Meyer unpub. data
Yellowfin tuna	*Thunnus albacares*	EPI Big	Meyer unpub. data
Yellowfin tuna	*Thunnus albacares*	EPI Big	Peng et al., 2013
Sailfish	*Istiophorus platypterus*	EPI Big	Thilakarathne & Attygalle, 2009
Skipjack tuna	*Katsuwonus pelamis*	EPI Big	Nichols et al., 2002
Humpback whale	*Megaptera novaeangliae*	Mammal	Waugh, Nichols, Schlabach, Noad, & Nash, 2014

Southern elephant seal	*Mirounga leonina*	Mammal	Bradshaw et al., 2003
Cape fur seals	*Arctocephalus pusillus*	Mammal	Arnould, Nelson, Nichols, & Oosthuizen, 2005
New Zealand sea lion	*Phocarctos hookeri*	Mammal	Meynier et al., 2008
Ringed seal	*Pusa hispida*	Mammal	Grahl-Nielsen et al., 2003
Harp seal	*Pusa groenlandica*	Mammal	Grahl-Nielsen et al., 2003
Snake eel	*Ophichthidae*	Small (Meso/EPI)	Piché, Iverson, Parrish, & Dollar, 2010
Black edged cusk eel	*Neobythites analis*	Small (Meso/EPI)	Piché, Iverson, Parrish, & Dollar, 2010
Abyssal cutthroat eel	*Meadia abyssalis*	Small (Meso/EPI)	Piché, Iverson, Parrish, & Dollar, 2010
Two-spined shrimp	*Heterocarpus ensifer*	Small (Meso/EPI)	Piché, Iverson, Parrish, & Dollar, 2010
Red-tipped shrimp	*Heterocarpus laevigatus*	Small (Meso/EPI)	Piché, Iverson, Parrish, & Dollar, 2010
Ribbonfish	*Lepidopus caudatus*	Small (Meso/EPI)	Nichols et al., 2002
Rudderfish	*Tubbia tasmanica*	Small (Meso/EPI)	Nichols et al., 2002
Medusa fish	*Centrolophus niger*	Small (Meso/EPI)	Nichols et al., 2002
Rudderfish	*Tubbia sp*	Small (Meso/EPI)	Nichols et al., 2002
Ribaldo	*Mora moro*	Small (Meso/EPI)	Nichols et al., 2002
Lantern fish	*Myctophidae*	Small (Meso/EPI)	Wang et al., 2019
Fangtooth lanternfish	*Anoplogaster cornuta*	Small (Meso/EPI)	Wang et al., 2019
Warmings lanternfish	*Ceratoscopelus warmingii*	Small (Meso/EPI)	Wang et al., 2019
Viperfish	*Chauliodus sp.*	Small (Meso/EPI)	Wang et al., 2019
Honeycomb scaly dragonfish	*Stomias affinis*	Small (Meso/EPI)	Wang et al., 2019

Bigscale deep-sea smelt	*Melanolagus bericoides*	Small (Meso/EPI)	Wang et al., 2019
Deep-water bristlemouth	*Cyclothone atraria*	Small (Meso/EPI)	Wang et al., 2019
Rattails	*Macrouridae*	Small (Meso/EPI)	Vlieg and Body 1988
Sabretooth fish	*Evermannellidae*	Small (Meso/EPI)	Wang et al., 2019
Wingfin snaggletooth	*Heterophotus ophistoma*	Small (Meso/EPI)	Wang et al., 2019
Bristlemouth	*Gonostomatidae*	Small (Meso/EPI)	Wang et al., 2019
Many-scaled Bigscale	*Melamphaes polylepis*	Small (Meso/EPI)	Wang et al., 2019
Alcock's boafish	*Stomias nebulosus*	Small (Meso/EPI)	Wang et al., 2019

Table S9: Metadata and habitat for potential prey items. All profiles listed below are the published means for the species, location or season. –D indicates demersal habitat use. See literature cited in Supplemental Materials for references.

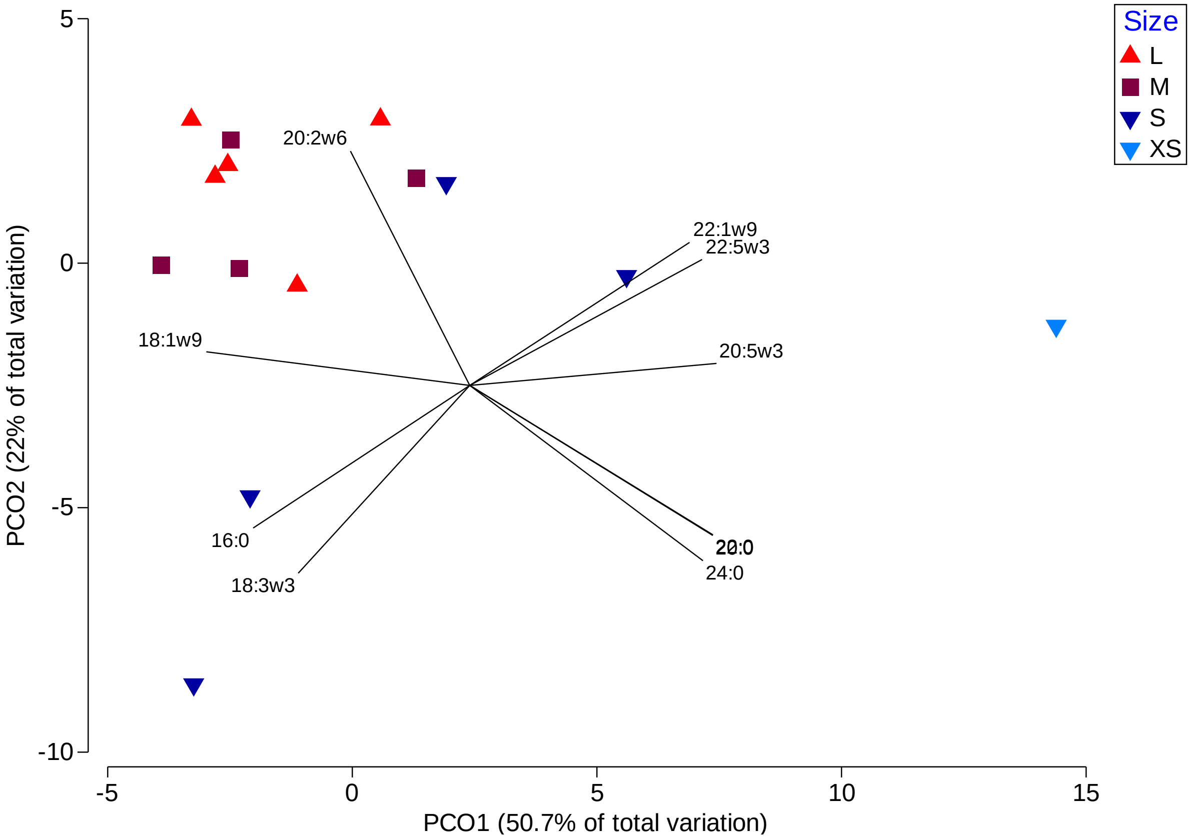

Figure S2: Principal Coordinate Analysis (PCA) of Cookiecutter Shark muscle FA profiles (% contribution) with vector overlays showing FAs with correlation values > 0.8. The results are displayed by size class.

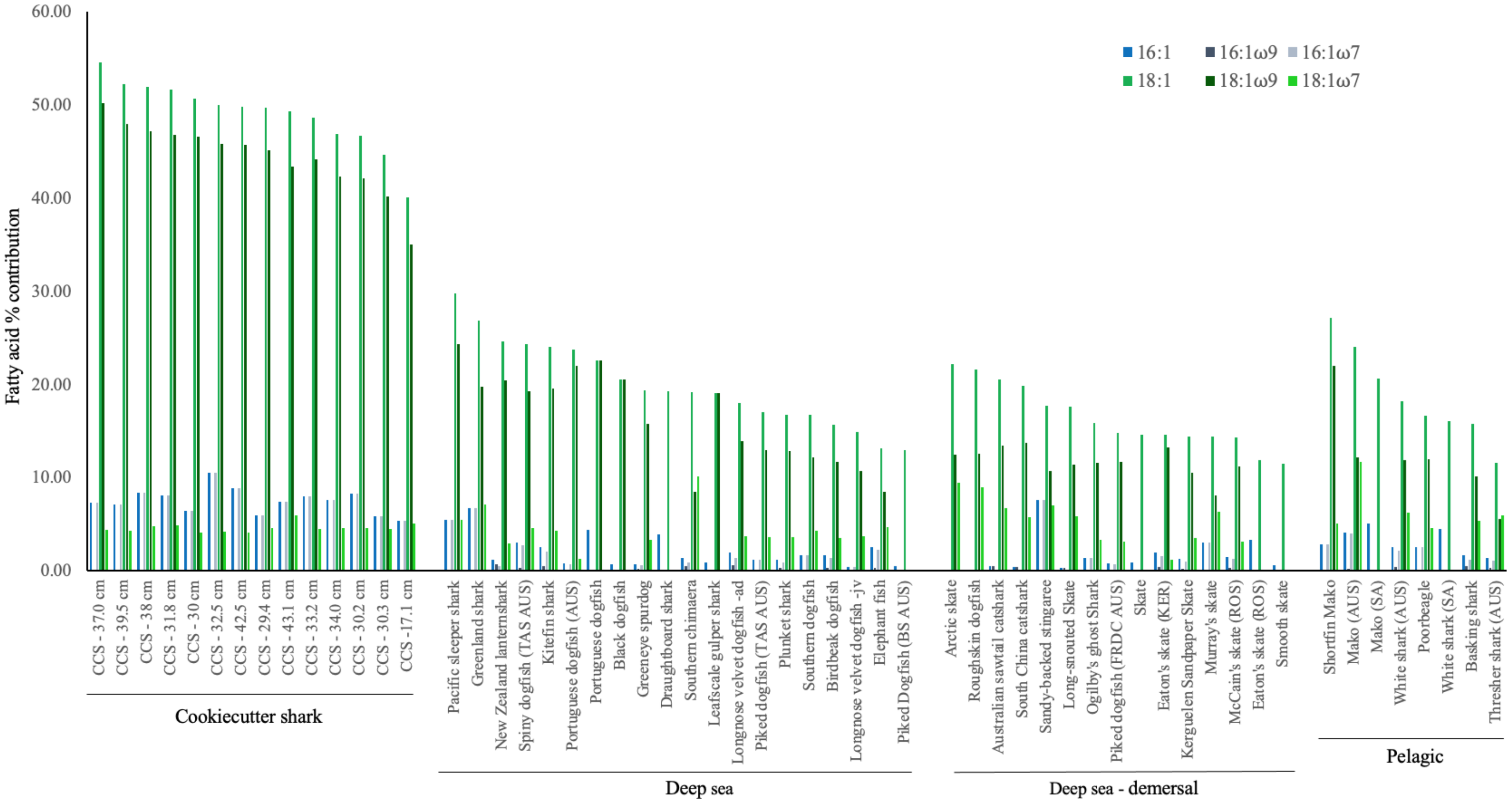

Figure S3: Muscle fatty acid percent contribution of 16:1 and 18:1 fatty acids for each Cookiecutter Shark, deep sea, deep sea-demersal and pelagic shark species. The Cookiecutter Shark label CCS is followed by the shark's total length (cm). The results are ordered by highest 18:1 per habitat group.

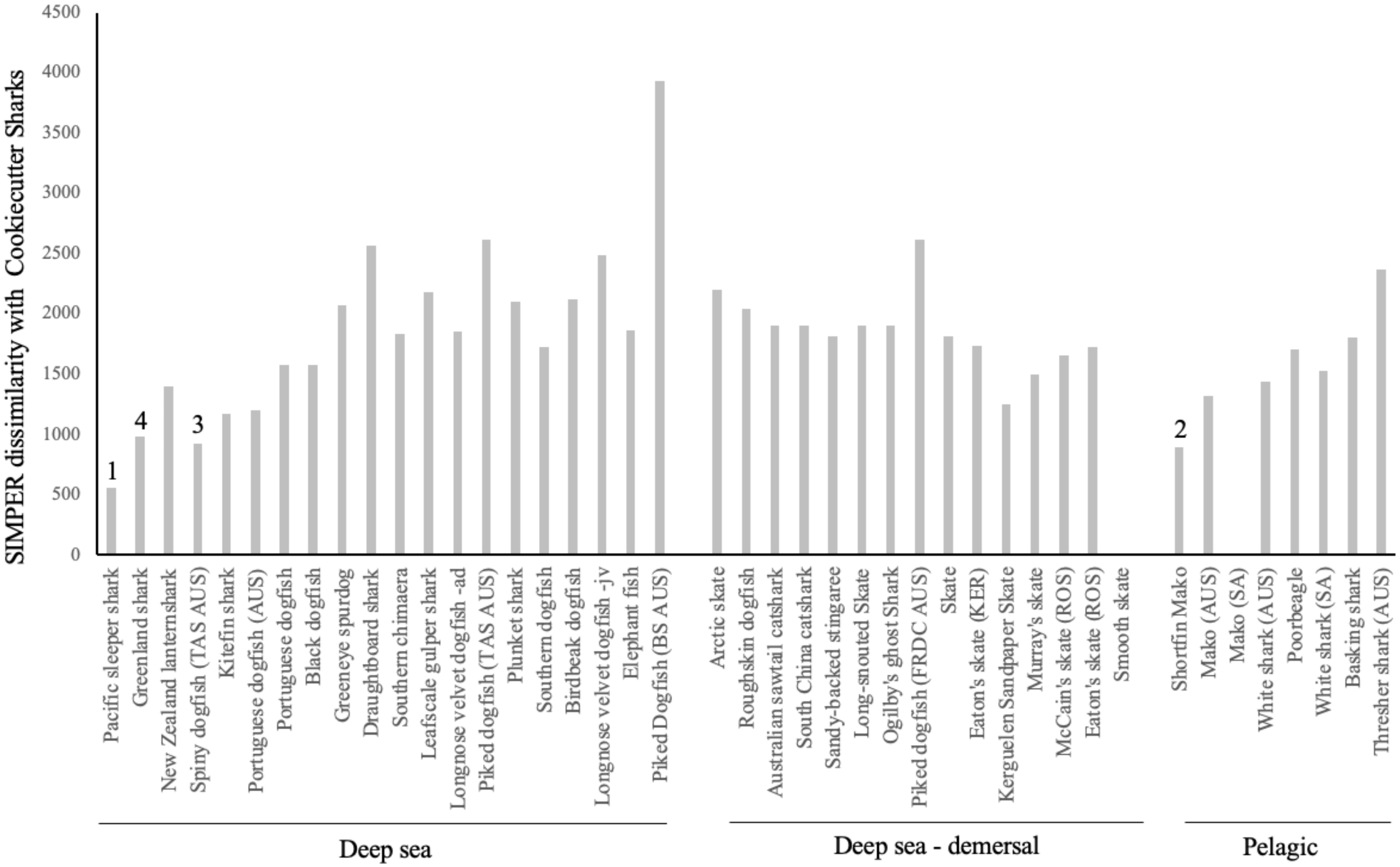

Figure S4: SIMPER dissimilarity scores for each species compared with the Cookiecutter Sharks. The species within each habitat group are ordered by most (left) to least (right) 18:1, as per figure S3. The four species most similar to the Cookiecutter Sharks are indicated by the 1-4 numbering within the figure.

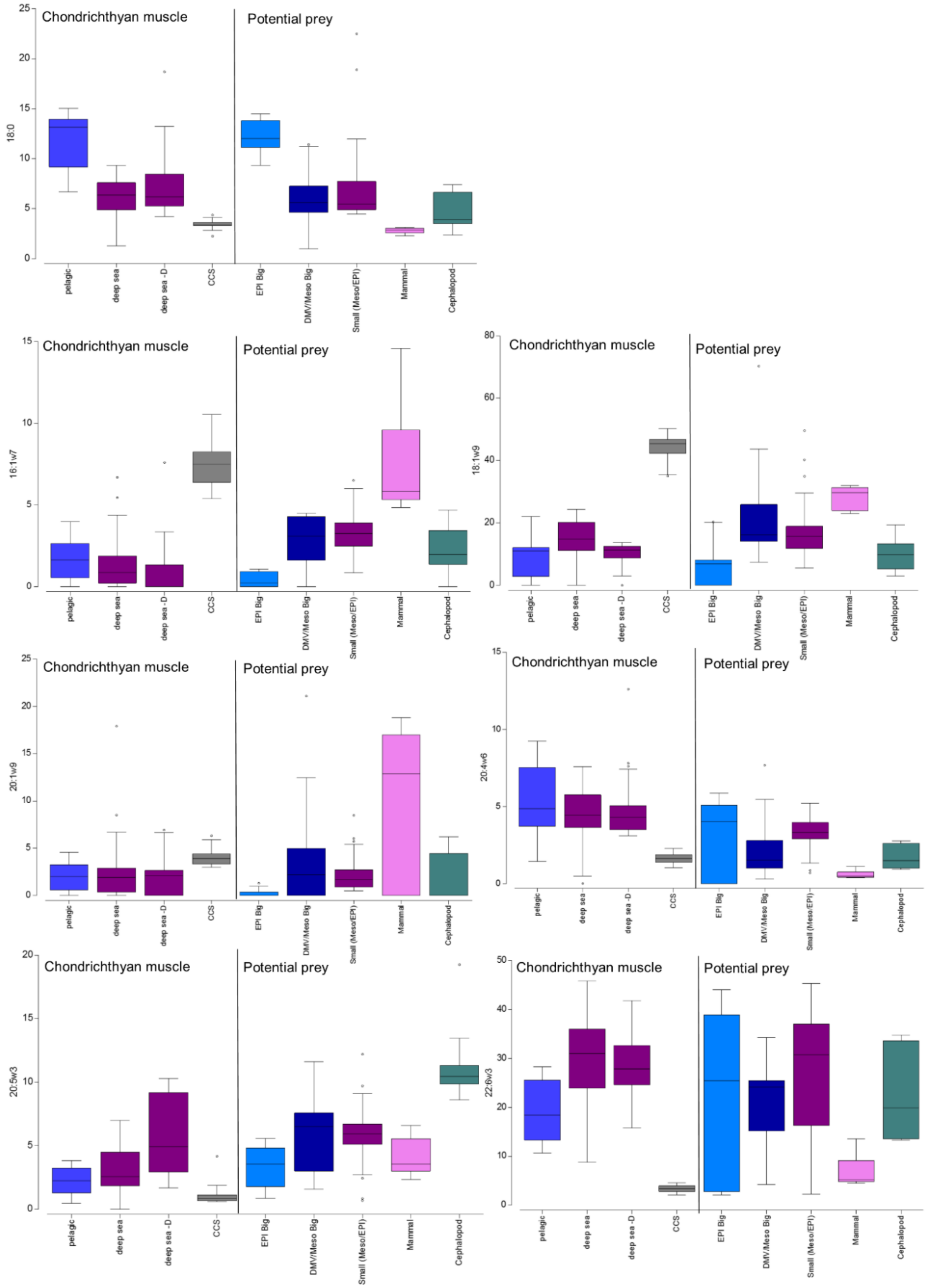

Figure S5: Select FAs (% contribution) from pelagic and deep sea chondrichthyans, Cookiecutter Sharks (CCS), and potential prey items.

Environmental DNA (eDNA)

DNA extraction and PCR amplification

DNA extractions were performed using the DNeasy Blood and Tissue Kit (Qiagen, USA) following the manufacturer's protocol with the following modification: for gastric fluid (not partially digested tissue), 200 mg of starting material was used to obtain sufficient yield of total DNA. Gastric fluid contents from the 11 stomachs were extracted in triplicate; four samples were taken from visible tissue present in three stomachs and were extracted in duplicate. Samples were randomized prior to extraction. DNA concentrations were determined using the Qubit dsDNA HS Assay (Invitrogen, CA, USA). In addition to the stomach samples, we also included negative controls (i.e., extraction blanks) and positive controls in our study. Negative controls included extraction blanks (n = 2) and PCR no-template controls (see below). Two different positive controls were included: 1) genomic DNA extracted from swordfish tissue (*Xiphias gladius*) and 2) a mock community with equimolar concentration of DNA from 10 species of bony fishes. Further description of the mock community is provided elsewhere (Port et al. 2016).

Before amplification with the tagged primers, all samples were amplified with untagged primers to determine if a band was present using the gel visualization. To check for inhibition in samples where a band was not present, the sample was diluted at 1:10 or 1:50 and amplified again. Based on the results of these tests (data not shown), samples were not diluted prior to the amplification with the tagged primers. Some extracts were also cleaned using a Zymo OneStep PCR Inhibitor Removal Kit (Zymo Research, CA, USA) prior to amplification if the extract was colored or if the band in the initial untagged PCR was faint. If a band was not present in the gel visualization, the sample was removed from further processing (n = 1 shark).

PCR reactions were carried out using 2 μL of DNA extract, 0.4 μL each of 10 μM forward and reverse primer, 10 μL of HotStarTaq Plus Master Mix (Qiagen, USA), and 7.2 μL of molecular-biology-grade water (Sigma-Aldrich, USA). Eight-strip PCR tubes with individually attached lids were used instead of 96-well plates to reduce cross-contamination between samples. Each DNA extract was amplified in triplicate, along with no template controls (NTCs) using molecular-biology-grade water in lieu of DNA template for each sample. Thermal conditions for PCR were 95 °C for 5 min followed by 40 cycles of 95 °C for 15 s, 55 °C for 30 s and 72 °C for 30 s. Triplicate PCR products were pooled and visualized on a 1.5% agarose gel stained with ethidium bromide to confirm the presence of the target band and no amplification in the NTCs. Pooled PCR products (and PCR products from tissue samples) were purified and size selected using the Agencourt AMPure XP bead system (Beckman Coulter, USA), and then quantified using the QUBIT dsDNA HS Assay.

Next generation sequencing and bioinformatic analysis

Tagged PCR products for gastric fluid samples yielding sufficient target DNA (n = 30 across 10 sharks) were pooled in equimolar concentration (20 ng DNA per sample) along with controls (n = 8) to create a single library. Note that of the 14 total sharks, three sharks did not have enough starting material for DNA extraction and amplicons from one shark were not visible on a gel. For extraction blanks or samples with less than 20 ng DNA, the entire amount of amplified DNA extract available was added to the pool. The concentration of the pool was 3.7 ng/μL, and 200 ng were used for library preparation with the KAPA low-throughput library prep kit with real-time library amplification protocol (KAPA Biosystems, USA). A NEXTFLEX DNA barcode (BIOO Scientific, USA) containing the Illumina adapter sequence was added during library preparation. Library size and concentration were confirmed using a Bioanalyzer

with High Sensitivity DNA assay (Agilent Technologies, USA). The library was sequenced on an Illumina MiSeq platform (250 bp, paired-end) at the Stanford Functional Genomics facility using a 20% PhiX spike-in control.

OTUs were compared to a local nucleotide database containing mitochondrial sequences from NCBI using BLAST+ (Camacho et al. 2009). This database—deposited in the Dryad Digital Repository—totaled 12,709 sequences and included the complete mitochondrial genomes as well partial 12S rRNA gene fragments of bony fishes (Actinopterygii), cartilaginous fishes (Chondrichthyes), true seals (Phocidae), sea lions (Otariidae), whales (Cetacea), marine dolphins (Delphinidae), sea otters (*Enhydra*) and birds (Aves) (sequences downloaded September 2014). Default BLAST parameters were used except for the following modifications: e-value = 1e-20, percent identity = 98%, and word size = 24. Taxonomy was assigned to the most specific rank possible (generally family or genus given resolution of 12S primers) using the lowest common ancestor (LCA) algorithm in MEGAN v5.5.3 (default settings except for: min score = 150, top percent = 2) (Huson and Weber 2013). We removed OTUs classified as non-marine vertebrates (e.g., *Homo sapiens*, *Gallus gallus*) as well as unassigned sequences and reads annotated as chimeras.

To account for uneven sequencing depths across samples, we rarefied each sample (stomachs and positive controls) to 100,000 reads using the “rrarefy” function in the R package vegan (Oksanen et al. 2010). We chose 100,000 as 28 out of the 30 stomach samples had > 100,000 reads. The two stomach samples with less than 100,000 (samples 6C and 7A with 839 and 3,256 reads respectively) had the majority of reads assigned to cookie-cutter shark or had no matches to the local 12S database; they were removed from subsequent analyses. False positive taxonomic assignments and low-frequency noise were removed from the dataset using a

restrictive approach in which only taxa with a minimum copy threshold of 10 reads per sample in at least two out of the three replicates for a given stomach were considered. A conservative threshold of 10 reads was based on an analysis of the positive controls and blanks. In the swordfish tissue samples and artificial community, taxa not expected to be present (i.e., cross-contamination) had no more than 5 reads. Sequence counts were analyzed in terms of presence/absence as well as relative abundance. Given biases associated with PCR amplification, sequencing, rarefaction, etc., our analysis was semi-quantitative. Rarefied read counts were binned on an order of magnitude scale (i.e., <100, <1000, <10,000 and <100,000).

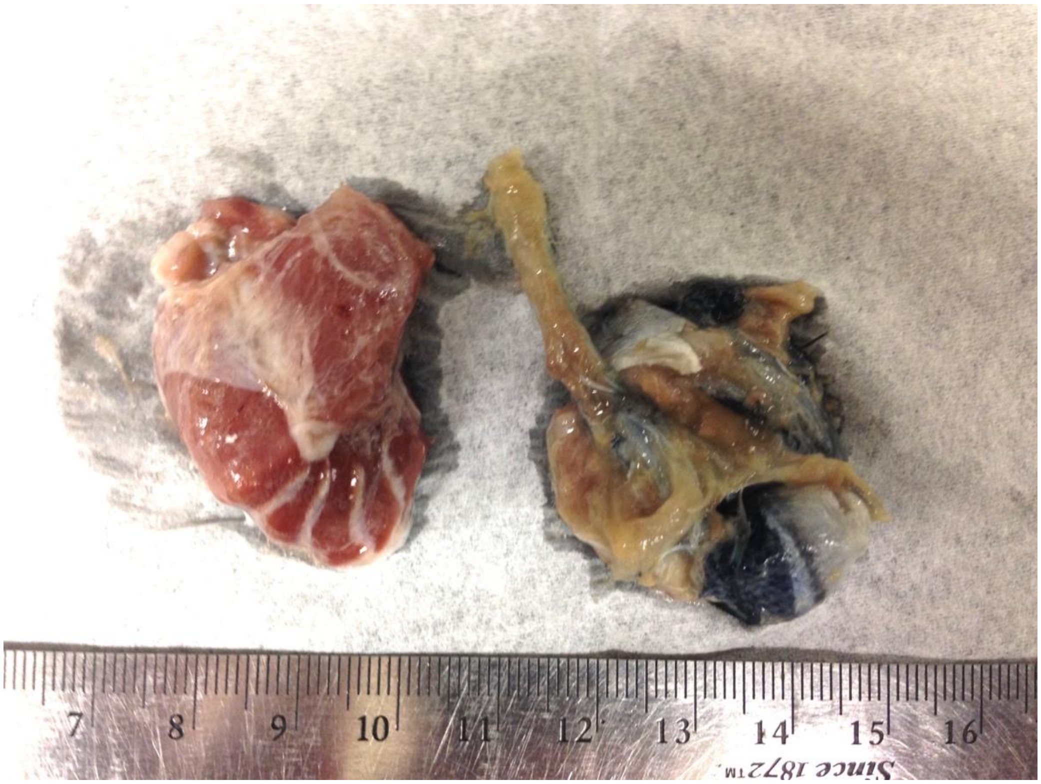

Figure S6: Tissue contents extracted from two Cookiecutter Shark stomachs. Sanger sequencing with the 12S primers identified these tissue samples as (a) *Thunnini* (tunas) and (b) *Cololabis saira* (Pacific saury).

Literature cited

Arnould, J. P. Y., Nelson, M. M., Nichols, P. D., & Oosthuizen, W. H. (2005). Variation in the fatty acid composition of blubber in Cape fur seals (*Arctocephalus pusillus pusillus*) and the implications for dietary interpretation. Journal of Comparative Physiology B, 175(4), 285–295.

Bradshaw, C. J. A., Hindell, M. A., Best, N. J., Phillips, K. L., Wilson, G., & Nichols, P. D. (2003). You are what you eat: describing the foraging ecology of southern elephant seals (*Mirounga leonina*) using blubber fatty acids. Proceedings of the Royal Society of London. Series B: Biological Sciences, 270(1521), 1283–1292.

Budge, S. M., Iverson, S. J., Bowen, W. D., & Ackman, R. G. (2002). Among-and within-species variability in fatty acid signatures of marine fish and invertebrates on the Scotian Shelf, Georges Bank, and southern Gulf of St. Lawrence. Canadian Journal of Fisheries and Aquatic Sciences, 59(5), 886–898.

Camacho C, Coulouris G, Avagyan V, Ma N, Papadopoulos J, Bealer K, Madden TL (2009) BLAST+: architecture and applications. BMC bioinformatics 10: 421

Carlisle AB, Goldman KJ, Litvin SY, Madigan DJ, Bigman JS, Swithenbank AM, Kline TC, Block BA (2015) Stable isotope analysis of vertebrae reveals ontogenetic changes in habitat in an endothermic pelagic shark. Proceedings of the Royal Society B-Biological Sciences 282: 20141446

Choy CA, Popp BN, Hannides CC, Drazen JC (2015) Trophic structure and food resources of epipelagic and mesopelagic fishes in the North Pacific Subtropical Gyre ecosystem inferred from nitrogen isotopic compositions. Limnology and oceanography 60: 1156-1171

Davidson, B., Sidell, J., Rhodes, J., & Cliff, G. (2011). A comparison of the heart and muscle total lipid and fatty acid profiles of nine large shark species from the east coast of South Africa. Fish Physiology and Biochemistry, 37(1), 105–112. doi:10.1007/s10695-010-9421-8

Dunstan, G. a, Sinclair, A. J., O'Dea, K., & Naughton, J. M. (1988). The lipid content and fatty acid composition of various marine species from southern Australian coastal waters. Comparative Biochemistry and Physiology Part B: Comparative Biochemistry, 91(1), 165–169. doi:10.1016/0305-0491(88)90130-7

Field, I. C., Nichols, P. D., Bradshaw, C. J. A., & Hindell, M. A. (2009). Blubber fatty acid profiles indicate dietary resource partitioning between adult and juvenile southern elephant seals. Marine Ecology Progress Series, 384, 303–312.

Gould P, Ostrom P, Walker W (1997) Trophic relationship of albatrosses associated with squid and large-mesh drift-net fisheries in the North Pacific Ocean. Canadian Journal of Zoology 75: 549 - 562

Grahl-Nielsen, O., Andersen, M., Derocher, A. E., Lydersen, C., Wiig, Ø., & Kovacs, K. M. (2003). Fatty acid composition of the adipose tissue of polar bears and of their prey: ringed seals, bearded seals and harp seals. Marine Ecology Progress Series, 265, 275–282.

Graham BS (2007) Trophic dynamics and movements of tuna in tropical Pacific Ocean inferred from stable isotope analyses. Ph.D. University of Hawaii at Manoa.

Graham BS, Grubbs D, Holland K, Popp BN (2007) A rapid ontogenetic shift in the diet of juvenile yellowfin tuna from Hawaii. Marine Biology 150: 647-658

Guil-Guerrero, J. L., Venegas-Venegas, E., Rincón-Cervera, M. Á., & Suárez, M. D. (2011).

Fatty acid profiles of livers from selected marine fish species. Journal of Food Composition and Analysis, 24(2), 217–222.

Huson DH, Weber N (2013) Microbial community analysis using MEGAN Methods in enzymology. Elsevier, pp 465-485

Jayasinghe, C., Gotoh, N., & Wada, S. (2003). Variation in lipid classes and fatty acid composition of salmon shark (*Lamna ditropis*) liver with season and gender. Comparative Biochemistry and Physiology - B Biochemistry and Molecular Biology, 134(2), 287–295. doi:10.1016/S1096-4959(02)00268-3

Jo, H.-S., Yeon, I., Lim, C., Hanchet, S. M., Lee, D.-W., & Kang, C.-K. (2013). Fatty acid and stable isotope analyses to infer diet of Antarctic toothfish caught in the southern Ross Sea. CCAMLR Science, 20, 21–36.

McMeans, B. C., Arts, M. T., & Fisk, A. T. (2012). Similarity between predator and prey fatty acid profiles is tissue dependent in Greenland sharks (*Somniosus microcephalus*): Implications for diet reconstruction. Journal of Experimental Marine Biology and Ecology, 429, 55–63. doi:10.1016/j.jembe.2012.06.017

Meynier, L., Morel, P. C. H., Mackenzie, D. D. S., MacGibbon, A., Chilvers, B. L., & Duignan, P. J. (2008). Proximate composition, energy content, and fatty acid composition of marine species from Campbell Plateau, New Zealand. New Zealand Journal of Marine and Freshwater Research, 42(4), 425–437.

Nichols, P. D., Mooney, B. D., & Elliott, N. G. (2002). Nutritional value of Australian seafood II: Factors affecting oil composition of edible species.

Økland, H. M. W., Stoknes, I. S., Remme, J. F., Kjerstad, M., & Synnes, M. (2005). Proximate

composition, fatty acid and lipid class composition of the muscle from deep-sea teleosts and elasmobranchs. Comparative Biochemistry and Physiology - B Biochemistry and Molecular Biology, 140(3), 437–443. doi:10.1016/j.cbpc.2004.11.008

Oksanen J, Blanchet FG, Kindt R, Legendre P, O’hara R, Simpson GL, Solymos P, Stevens MHH, Wagner H (2010) Vegan: community ecology package. R package version 1.17-4. http://cran. r-project. org>. Acesso em 23: 2010

Parry MP (2003) The trophic ecology of two ommastrephid squid species, *Ommastrephes bartamii* and *Sthenoteuthis oualaniensis*, in the North Pacific sub-tropical gyre. Ph.D., Manoa

Parry MP (2008) Trophic variation with length in two ommastrephid squids, *Ommastrephes bartramiii* and *Sthenoteuthis oualaniensis*. Marine Biology 153: 249-256

Peng, S., Chen, C., Shi, Z., & Wang, L. (2013). Amino Acid and Fatty Acid Composition of the Muscle Tissue of Yellowfin Tuna (*Thunnus albacares*) and Bigeye Tuna (Thunnus Obesus). Journal of Food and Nutrition Research, 1(4), 42–45. doi:10.12691/jfnr-1-4-2

Pethybridge, H., Daley, R., Virtue, P., & Nichols, P. (2010). Lipid composition and partitioning of deepwater chondrichthyans: Inferences of feeding ecology and distribution. Marine Biology, 157(6), 1367–1384. doi:10.1007/s00227-010-1416-6

Pethybridge, H. R., Parrish, C. C., Bruce, B. D., Young, J. W., & Nichols, P. D. (2014). Lipid, fatty acid and energy density profiles of white sharks: Insights into the feeding ecology and ecophysiology of a complex top predator. PLoS ONE, 9(5). doi:10.1371/journal.pone.0097877

Piché, J., Iverson, S. J., Parrish, F. A., & Dollar, R. (2010). Characterization of forage fish and

invertebrates in the Northwestern Hawaiian Islands using fatty acid signatures: species and ecological groups. Marine Ecology Progress Series, 418, 1–15.

Port JA, O'Donnell JL, Romero-Maraccini OC, Leary PR, Litvin SY, Nickols KJ, Yamahara KM, Kelly RP (2016) Assessing vertebrate biodiversity in a kelp forest ecosystem using environmental DNA. Molecular ecology 25: 527-541

Remme JF, Larssen WE, Bruheim I, Sæbø PC, Sæbø A, Stoknes IS (2006). Lipid content and fatty acid distribution in tissues from Portuguese dogfish, leafscale gulper shark and black dogfish. Comparative Biochemistry and Physiology - B Biochemistry and Molecular Biology, 143(4), 459–464. doi:10.1016/j.cbpb.2005.12.018

Schaufler, L., Heintz, R., Sigler, M., & Hulbert, L. (2005). Fatty acid composition of sleeper shark (*Somniosus pacificus*) liver and muscle reveals nutritional dependence on planktivores. Ices Cm.

Thilakarathne, L. a D. S., & Attygalle, M. V. E. (2009). Lipid composition of skin and muscle of the Indo-Pacific sailfish , *lstiophorus platypterus*, 14(1), 161–166.

Vlieg, P., Murray, T., & Body, D. R. (1993). Nutritional data on six oceanic pelagic fish species from New Zealand waters. Journal of Food Composition and Analysis, 6(1), 45–54.

Wang, F., Wu, Y., Chen, Z., Zhang, G., Zhang, J., Zheng, S., & Kattner, G. (2019). Trophic Interactions of Mesopelagic Fishes in the South China Sea Illustrated by Stable Isotopes and Fatty Acids . Frontiers in Marine Science . Retrieved from https://www.frontiersin.org/article/10.3389/fmars.2018.00522

Waugh, C. A., Nichols, P. D., Schlabach, M., Noad, M., & Nash, S. B. (2014). Vertical

distribution of lipids, fatty acids and organochlorine contaminants in the blubber of southern hemisphere humpback whales (*Megaptera novaeangliae*). Marine Environmental Research, 94, 24–31.

Wheeler, S. C., & Morrissey, M. T. (2003). Quantification and distribution of lipid, moisture, and fatty acids of West Coast albacore tuna (*Thunnus alalunga*). Journal of Aquatic Food Product Technology, 12(2), 3–16.

Witteveen BH, Worthy GAJ, Roth JD (2009) Tracing migratory movements of breeding North Pacific humpback whales using stable isotope analysis. Marine Ecology Progress Series 393: 173-183
